# PADI2‐Catalyzed MEK1 Citrullination Activates ERK1/2 and Promotes IGF2BP1‐Mediated SOX2 mRNA Stability in Endometrial Cancer

**DOI:** 10.1002/advs.202002831

**Published:** 2021-01-29

**Authors:** Teng Xue, Xiaoqiu Liu, Mei Zhang, Qiukai E, Shuting Liu, Maosheng Zou, Ying Li, Zhinan Ma, Yun Han, Paul Thompson, Xuesen Zhang

**Affiliations:** ^1^ State Key Laboratory of Reproductive Medicine Nanjing Medical University Nanjing Jiangsu 211166 China; ^2^ Key Laboratory of Pathogen Biology of Jiangsu Province Department of Microbiology Nanjing Medical University Nanjing Jiangsu 211166 China; ^3^ Department of Obstetrics Dalian Municipal Maternal and Infant Health Care Hospital Dalian Liaoning 116000 China; ^4^ Department of Obstetrics and Gynecology Yangzhou Maternal and Child Health Hospital Yangzhou University Yangzhou Jiangsu 225009 China; ^5^ Department of Obstetrics and Gynecology The Second Affiliated Hospital of Nantong University Nantong Jiangsu 226001 China; ^6^ Department of Biochemistry and Molecular Pharmacology University of Massachusetts Medical School Worcester MA 01655 USA

**Keywords:** citrullination, endometrial cancer, insulin‐like growth factor‐II binding protein 1, MEK1, peptidylarginine deiminase II, RNA stability

## Abstract

Peptidylarginine deiminase II (PADI2) converts positively charged arginine residues to neutrally charged citrulline, and this activity has been associated with the onset and progression of multiple cancers. However, a role for PADI2 in endometrial cancer (EC) has not been previously explored. This study demonstrates that PADI2 is positively associated with EC proregression. Mechanistically, PADI2 interacting and catalyzing MEK1 citrullination at arginine 113/189 facilitates MEK1 on extracellular signal‐regulated protein kinases 1/2 (ERK1/2) phosphorylation, which activates insulin‐like growth factor‐II binding protein 1 (IGF2BP1) expression. Furthermore, RNA immunoprecipitation (RIP) and RNA stability analyses reveal that IGF2BP1 binds to the m^6^A sites in *SOX2*‐3′UTR to prevent *SOX2* mRNA degradation. Dysregulation of IGF2BP1 by PADI2/MEK1/ERK signaling results in abnormal accumulation of oncogenic SOX2 expression, therefore supporting the malignant state of EC. Finally, PADI2 gene silencing, inhibiting MEK1 citrullination by PADI2 inhibitor, or mutation of MEK1 R113/189 equally inhibits EC progression. These data demonstrate that PADI2‐catalyzed MEK1 R113/189 citrullination is a critical diver for EC malignancies and suggest that targeting PADI2/MEK1 can be a potential therapeutic approach in patients with EC.

## Graphical abstract

Peptidylarginine deiminase II (PADI2) is highly expressed in endometrial cancer (EC) and promotes its progression. Mechanistically, PADI2‐catalyzed MEK1 citrullination at arginine 113 and 189 promotes MEK1 on ERK1/2 phosphorylation, leading to an increase of insulin‐like growth factor‐II binding protein 1 (IGF2BP1) expression. Aberrant activation of IGF2BP1 stabilizes oncogenic Sox2 mRNA, therefore supporting the malignant state of EC.

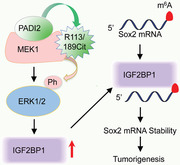

## 1 Introduction

Endometrial cancer (EC), originated from endometrium, is one of the three most common prevalent gynecological malignancies worldwide.^[^ ^1^ ^]^ Currently, the principle treatment is mainly hysterectomy in the early stage of EC, and surgery with other adjuvant therapy in the advanced stage.^[^ ^2^ ^]^ However, EC cells in the advanced stage are highly invasive and easily metastatic, accompanied by a low survival rate and poor prognosis.^[^ ^1^ ^]^ Even for young women with early‐stage EC, conventional surgery brings devastating consequences for fertility to these patients.^[^ ^3^ ^]^ Therefore, it is of great significance to further understand the key mechanisms driving endometrial carcinogenesis and find novel molecular prognostic biomarkers, as well as promising therapeutic target for EC.

The Ras/Raf/MEK/ERK mitogen‐activated protein kinase (MAPK) signaling cascade is one of the best‐characterized signaling module that plays a significant role in multiple physiological and pathological behaviors,^[^ ^4^ ^]^ and aberrant activation of this pathway is perhaps the most important oncogenic driver of human cancers.^[^ ^5^ ^]^ As a central component in the MAPK signaling cascade, MEK1/2 are phosphorylated and activated by upstream Raf kinase at the Ser218 and Ser222 residues, which, in turn, exclusively catalyze the phosphorylation and activation of downstream nuclear transcription factor, extracellular signal‐regulated protein kinases 1/2 (ERK1/2). Therefore, this pivotal intersectional position of MEK1/2 makes it an attractive antitumor target in cancer therapy, including EC.^[^ ^6^ ^]^ Currently, several MEK inhibitors have been developed, and some have shown remarkable potency and selectivity during clinical evaluation.^[^ ^7^ ^]^ However, patients treated with RAF or MEK inhibitors frequently develop drug resistance within several months.^[^ ^8^ ^]^ Given this situation, researchers continue to pursue approaches that can reverse drug resistance, and develop innovative combination strategies with other targeted therapeutics to overcome these challenges.^[^ ^9^ ^]^

The peptidylarginine deiminases (PADIs) contain five family members, namely PADI1‐4 and PADI6. Except for PADI6, which does not contain the enzymatic activity and is restrictedly expressed in ovary,^[^ ^10^ ^]^ all the other PADIs can post‐translationally convert positively charged arginine residues to the neutral, non‐coded residue citrulline in substrate proteins, termed citrullination or deamination.^[^ ^11^, ^12^ ^]^ Recently, there is growing evidence from basic to clinical studies that supports the crucial roles of PADIs associated with the onset and progression of cancers. Indeed, the higher PADIs expression in a wide range of human malignant cancers compared to healthy tissue,^[^ ^13^, ^14^ ^]^ together with the fact that synthetic PADI inhibitors can kill a selection of cancerous cell lines, strongly suggests that PADIs‐catalyzed citrullination may play important roles in tumorigenesis.^[^ ^15^, ^16^ ^]^ Such hypothesis has been supported by the rapidly accumulated studies, demonstrating that PADIs‐catalyzed protein citrullination can alter cell signaling, cell differentiation, and epithelial to mesenchymal transition in multiple human cancer cells.^[^ ^14^, ^17^ ^]^ However, the role of PADIs and PADIs‐catalyzed citrullination in the tumorigenesis of EC is totally unclear. In this study, we sought to elucidate the mechanism of peptidylarginine deiminase II (PADI2)‐catalyzed MEK1 citrullination in progression of EC and propose new treatment concepts for EC by targeting PADI2.

## 2 Results

### 2.1 PADI2 Expression is Positively Associated with Endometrial Carcinoma Proregression

**Figure 1:**
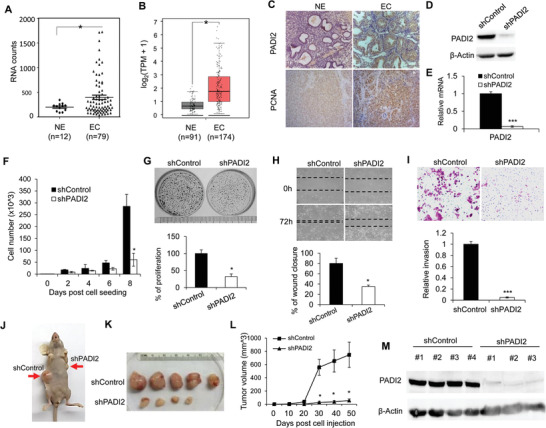
PADI2 expression is positively associated with endometrial carcinoma proregression. A) Analysis of PADI2 mRNA levels in human endometrial cancer tissues compared to normal endometrium tissues in GEO profile (https://www.ncbi.nlm.nih.gov /geoprofiles/?term = 100098900), and B) in GEPIA database. C) Expression levels of PADI2 and PCNA were examined by immunohistochemistry staining in representative sections from EC patients (*n* = 8) and normal endometrium section (*n* = 8), original magnification, × 40. D) Analysis for PADI2 protein, and E) mRNA expression in PADI2 stable knockdown ISI cells. *β*‐actin served as loading control. F) PADI2 knockdown or shRNA control ISI cells were cultured in regular medium, at indicated times, and cell numbers were counted under light microscope. G) Representative images of crystal violet staining of cells in 35 mm‐wells (top) and quantification (bottom) of colony formation ability of PADI2 KD ISI cells and control cells. H) Representative images at indicated hours after scratching (top) and quantification (bottom) for wound healing assay in PADI2 KD ISI cells and control cells. I) Representative images (top) and quantification (bottom) for transwell assay in PADI2 KD ISI cells and control cells. J) A reprehensive nude mouse injected with shRNA control ISI cells on the left flank (shControl) and stable PADI2 KS ISI cellson the right flank (shPADI2) at the experimental endpoint. K) Dissected tumors collected from nude mice (*n* = 5). L) The volume of tumors at indicated time after implantation of PADI2 KD ISI cells and control cells in nude mice. M) Expression of PADI2 in tumor lysates. *β*‐actin served as a loading control. Results are presented as mean ± SEM, *n* = 3 (D–I). *p < 0.05, ***p < 0.001.

We first evaluated the mRNA expression of five PADIs family members in endometrial cancer tissues in GEPIA, a web server for cancer and normal gene expression profiling and interactive analyses (http://gepia.cancer-pku.cn),^[^ ^18^ ^]^ and found a relatively abundant PADI2 expression compared to the other PADIs. Note: PADI4 and PADI6 mRNA levels are below the detection limit (Figure S1A, Supporting Information). To determine the clinical significance of PADI2 in endometrial carcinoma, we first examined PADI2 mRNA expression levels in clinical human endometrial cancer tissue microarray (micro‐dissected frozen uterine tumors) using the publicly GEO profile 100098900 from dataset GSE17025.^[^ ^19^ ^]^ As shown in **Figure** **1**A, PADI2 transcript levels were significantly elevated in EC tissue compared to that in the inactive endometrium from the postmenopausal controls (*p* = 0.041). Additionally, we also queried the PADI2 mRNA in GEPIA and observed an increase in PADI2 levels in EC tissue compared to normal endometrium (*p*‐value cutoff = 0.01; Figure 1B). To confirm these findings, we compared PADI2 protein levels in clinical endometrial tumor tissues using immunohistochemistry. PADI2 staining was strongly positive in seven out of eight endometrial tumor sections, while less positive in the eight normal endometrium sections (representative image shown in Figure 1C). Correspondingly, immunohistochemical analysis revealed stronger expression of the cell proliferation marker PCNA in endometrial tumor sections. To further study the role of PADI2 in endometrial carcinogenesis, we generated PADI2‐depleted human endometrial cancer Ishikawa (ISI) cells using a lentiviral approach. PADI2 knockdown efficiency was confirmed via western blot and qRT‐PCR (Figure 1D,E; Figure S2A, Supporting Information). PADI2 depletion suppressed ISI cell proliferation in 2D monolayer cultures (Figure 1F,G; Figure S2B, Supporting Information). We next examined the effect of PADI2 knockdown on ISI cell migration using wound‐healing assays. Results showed that PADI2 depletion reduced the ability of ISI cells to migrate into the wound compared to the controls (Figure 1H; Figure S2C, Supporting Information). We then performed transwell invasion assay across a matrigel membrane and found that PADI2 depletion decreased the ability of ISI cells to migrate across the gel matrix into the adjacent chamber (Figure 1I; Figure S2D, Supporting Information). We also evaluated the effect of PADI2 knockdown on cell proliferation, migration, invasion, and spheroid growth in another endometrial carcinoma cell line, ECC‐1. As expected, PADI2 silencing inhibited ECC‐1 cell proliferation and invasion (Figures S1B–E and S2B–E, Supporting Information). Furthermore, we confirmed the in vitro phenotype of ISI cells upon PADI2 knockdown in xenograft mouse model. The mice bearing PADI2 knockdown cells all exhibited smaller tumors than the mice with shRNA control cells (Figure 1J,K), and this difference became significant 30 days post cell injection (Figure 1L). We also confirmed the knockdown efficiency of PADI2 in established tumors by western blot. As expected, the tumors in mice inoculated with PADI2 KD cell lines had diminished expression of PADI2 (Figure 1M). These results suggested that PADI2 is required for proliferation, migration, and invasion of endometrial carcinoma cells.

### 2.2 IGF2BP1 is a Downstream Target for PADI2 and Mediates Endometrial Carcinoma Progression

**Figure 2:**
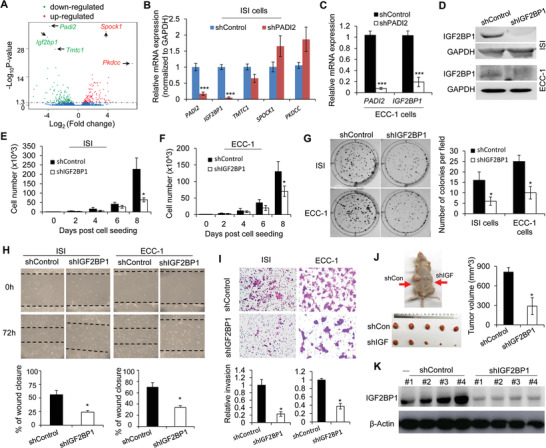
IGF2BP1 is a downstream target for PADI2 and mediates endometrial carcinoma progression. A) Volcano plot showing enrichment of dysregulated target genes determined by RNA‐seq in PADI2‐knockdown versus control ISI cells. The relative expression levels for each gene depicted as Log_2_ expression fold changes (FC) plotted against –Log_10_ (*p* value). Differentially expressed genes (FC > 1.0 or < −1.0 and *p* value < 0.01) are shown. The positions of PADI2 and genes used for validation are also indicated. B) qRT‐PCR analysis of *PADI2, IGF2BP1, TMTC1, SPOCK1*, and *PKDCC* mRNA levels upon PADI2 silencing in ISI cells and C) *PADI2, IGF2BP1* mRNA levels upon PADI2 silencing in ECC‐1 cells. D) Western blot validation of IGF2BP1 knockdown in both ISI cells and ECC‐1 cells. GAPDH served as loading control. E) PADI2 knockdown or shRNA control ISI cells and F) ECC‐1 cells were cultured in regular medium, at indicated times, cell numbers were counted under light microscope. G) Representative images of crystal violet staining of cells (left) and quantification (right) of colony formation upon IGF2BP1 stable knockdown in ISI or ECC‐1 cells. H) Representative images at indicated hours after scratching (top) and quantification (bottom) for wound healing assay in IGF2BP1 KD ISI or ECC‐1 cells. I) Representative images (top) and quantification (bottom) for transwell assay in IGF2BP1 KD ISI or ECC‐1 cells. J) Left: Dissected tumors collected from nude mice (*n* = 5) injected with shRNA control ISI cells on the left flank (shControl), and stable IGF2BP1 depletion ISI cells on the right flank (shIGF) at the experimental endpoint. Right: The volume of tumors at indicated time after implantation of IGF2BP1 depletion cells or control cells in nude mice. K) Protein levels of IGF2BP1 in tumor lysates. *β*‐actin served as loading control. Results are presented as mean ± SEM, *n* = 3 (B–I). *p < 0.05.

To analyze the molecular mechanisms by which PADI2 induces endometrial carcinoma cell progression, we conducted RNA sequencing (RNA‐seq) in PADI2 knockdown and control ISI cells. With a threshold value of twofold expression change and a statistical significance of *p* < 0.005, the volcano plot showed the top three‐downregulated and two‐upregulated genes upon PADI2 knockdown in comparison to the control cells (**Figure** **2**A). We then validated the expression of these genes and successfully confirmed PADI2 (Figure 2B). Among the other four genes, insulin‐like growth factor‐II binding protein 1 (*IGF2BP1*) was confirmed to be the most downregulated (Figure 2B). These analyses indicated that IGF2BP1 may be the top candidate target for PADI2 in endometrial carcinoma cells. To further confirm this hypothesis, we tested the *IGF2BP1* expression in the other EC cell line. Results showed that *IGF2BP1* expression was also markedly inhibited upon PADI2 depletion in ECC‐1 cells (Figure 2C).

The oncofetal mRNA‐binding protein IGF2BP1 has the most conserved “oncogenic” role of the IGF2BP family in tumor‐derived cells,^[^ ^20^ ^]^ promoting a mesenchymal tumor cell phenotype characterized by elevated proliferation, migration, and invasion.^[^ ^21^, ^22^, ^23^ ^]^ Given that PADI2 regulates IGF2BP1 expression in EC cells, it is possible that depletion of IGF2BP1 may have the same effect on inhibiting EC cell progression as that of PADI2 knockdown. To test this, we first successfully established IGF2BP1 stable silencing ISI and ECC‐1 cells (Figure 2D). As expected, knockdown of IGF2BP1 inhibited EC cell proliferation (Figure 2E,F), colony formation (Figure 2G), migration (Figure 2H), and invasion (Figure 2I) in both cell lines. Further, the in vivo tumor xenograft mouse model also confirmed that the tumor inoculated with IGF2BP1 knockdown cells all exhibited smaller volume than the sides inoculated with control cells (Figure 2J). The knockdown efficiency of IGF2BP1 in established tumors was confirmed by western blot. The tumors in mice inoculated with IGF2BP1‐depleted cells had diminished levels of IGF2BP1expression (Figure 2K). The presented findings suggested that PADI2 and IGF2BP1 exhibit similar effects on promoting a pro‐proliferative and pro‐invasive signature in EC cells.

### 2.3 PADI2 Targets MEK1 for Citrullination, Leading to ERK1/2 Activation and IGF2BP1 Expression

**Figure 3:**
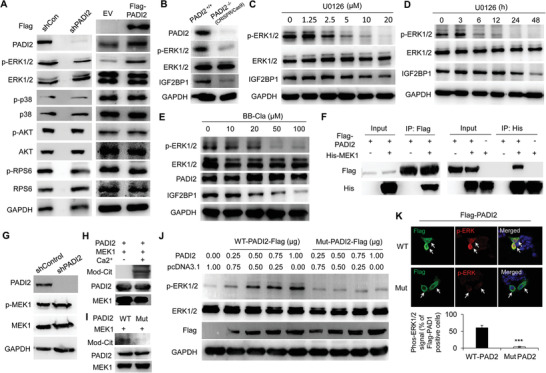
PADI2 targets MEK1 for citrullination and induces ERK1/2 activation and IGF2BP1 expression. A) Western blot analysis of PADI2, p‐ERK1/2, ERK1/2, p‐p38, p38, p‐AKT, AKT, p‐RPS6, and RPS6 upon PADI2‐depletion (left) or Flag‐tagged PADI2 overexpression (right) in ISI cells. The anti‐Flag antibody was used to confirm the exogenous expression of Flag‐tagged PADI2. GAPDH used as loading control. B) Western blot analysis of PADI2, p‐ERK1/2, ERK1/2, and IGF2BP1 in CRISPR/Cas9‐mediated PADI2 knockout ISI cells or control cells. GAPDH used as loading control. C) Western blot analysis of ISI cells treated with U0126 at indicated dose for 48 h, or D) 20 µm U0126 at indicated time using anti‐p‐ERK1/2, anti‐ERK1/2, anti‐IGF2BP1 antibodies. GAPDH served as loading control. E) Western blot analysis of ISI cells treated with BB‐Cl‐amidine using anti‐p‐ERK1/2, anti‐ERK1/2, anti‐PADI2, and anti‐IGF2BP1 antibodies. GAPDH served as loading control. F) Reciprocal co‐immunoprecipitation analysis of the interaction between PADI2 and MEK1 in HEK293 cells overexpressed Flag‐tagged PADI2 and His‐tagged MEK1. G) Western blot analysis of PADI2, p‐MEK1, MEK1, and GAPDH in PADI2 knockdown or control ISI cells. H) Citrullination of MEK1 by PADI2 in vitro in the presence or absence of Ca^2+^, or I) treated with wild type or catalytic inactive mutant PADI2. The reactions were assessed by western blot using anti‐Pan‐Cit, anti‐PADI2, and anti‐MEK1antibody. J) Western blot analysis of p‐ERK1/2, ERK1/2, Flag‐PADI2, and GAPDH in ISI cells overexpressed Flag‐tagged PADI2 (wild type or catalytic inactive mutant). K) Representative immunofluorescence images (top) and quantification (bottom) of co‐localization of PADI2 and p‐ERK1/2 in ISI cells overexpressing Flag‐PADI2 (WT or Mut). Arrows indicating signal positive cells. Results are presented as mean ± SEM, *n* = 3. *p <0.001.

Currently, it is not clear how PADI2 regulates IGF2BP1 expression. A variety of signaling pathways are activated in EC cells to regulate the target gene expression, including ERK MAPK signaling,^[^ ^4^, ^5^, ^24^ ^]^ and PI3K/AKT/mTOR.^[^ ^25^, ^26^ ^]^ To test whether PADI2 could also affect these signaling pathways in EC cells to regulate IGF2BP1 expression, we first performed western blot analyses on these kinases. As shown in **Figure** **3**A, PADI2 knockdown did not affect the activation of p38, AKT, or RPS6 in ISI cells. However, shRNA‐mediated PADI2 knockdown or CRISPR/Cas9‐mediated PADI2 depletion led to a decrease of ERK1/2 phosphorylation (Figure 3A,B; Figure S3, Supporting Information). Reversely, overexpression of PADI2 in ISI cells obviously promoted the phosphorylation level of ERK1/2, while having no effect on the activation of the other kinases (Figure 3A). To further test the hypothesis that PADI2 could affect IGF2BP1 expression through ERK1/2 activation, we treated ISI cells with the MEK1/2 inhibitor U0126 and then tested the IGF2BP1 expression. Results showed that U0126 globally suppressed ERK1/2 phosphorylation, and obviously inhibited IGF2BP1 expression in a dose‐ and time‐dependent manner (Figure 3C,D; Figure S4A, Supporting Information), suggesting that IGF2BP1 expression is dependent on PADI2‐regualted p‐ERK1/2 activation. Next, we tested whether inhibiting PADI2 enzymatic activity affects ERK1/2 activation and IGF2BP1 expression, using BB‐Cl‐amidine, a newly developed arginine‐based PADI2 inhibitor. Results showed that inhibiting PADI2 clearly inhibited ERK1/2 phosphorylation and IGF2BP1 expression (Figure 3E). The decreased expression of IGF2BP1 was also found in ISI cells with CRISPR/Cas9‐mediated PADI2 depletion, compared to the control cells (Figure 3B). These observations suggested that PADI2 activity might be required for regulating ERK signaling and the downstream IGF2BP1 expression.

The MAPK pathway represents a cascade of phosphorylation events including three pivotal kinases, namely Raf, MEK (MAP kinase kinase), and ERK (MAP kinase).^[^ ^27^, ^28^ ^]^ Given the striking correlation between PADI2 and ERK1/2 activation in mediating IGF2BP1 expression, we wondered whether PADI2 may target MEK1 to regulate ERK1/2 phosphorylation, and thus affect IGF2BP1 expression. To test this hypothesis, we first carried out co‐immunoprecipitation analysis and showed a reciprocal binding between ectopically expressed PADI2 and MEK1 in HEK293 cells (Figure 3F). Moreover, we performed immunofluorescence analysis in ISI cells transfected with Flag‐tagged PADI2 and His‐tagged MEK1 plasmids and found a strong co‐localization between exogenously expressed PADI2 and MEK1 (Figure S4B, Supporting Information). These results demonstrated that PADI2 could physically associate with MEK1. Then, we confirmed the phosphorylated MEK1 was not affected in PADI2 knockdown cells, suggesting that PADI2 does not affect Raf‐mediated MEK1 activation (Figure 3G). The findings that PADI2 interacted with MEK1 and that inhibition of PADI2 decreased phosphorylation of ERK1/2 (but not MEK1) suggested that PADI2 might target MEK1 to facilitate MEK1 enzyme activity, thus promoting the subsequent phosphorylation of ERK1/2 by MEK1.

### 2.4 PADI2 Targets MEK1 Specifically at Arg113/189 for Citrullination and Induces ERK1/2 Activation

Our previous study has shown that PADI1 can regulate to function on MEK1 via citrullination and therefore affect MEK1 enzyme activity in breast cancer cells,^[^ ^29^ ^]^ therefore, we wondered whether PADI2 could play the similar role. To test this hypothesis, His‐tagged MEK1 was purified, treated with recombinant PADI2, and the resolved proteins were then probed with an antibody that is reactive with citrullinated proteins (anti‐Pan‐Cit). Results showed that the anti‐Pan‐Cit antibody was reactive with an appropriately sized band (45 kDa) from the MEK1 purification and was not reactive with proteins without Ca^2+^ (Ca^2+^ is an essential ion for PADI2 catalyzed reaction) treatment (Figure 3H), or treated with catalytic inactive form of PADI2 (Mut) (Figure 3I), raising a possibility that MEK1 is a target for PADI2‐mediated citrullination. To further investigate whether MEK1 citrullination directly activates MEK1‐catalyzed ERK1/2 phosphorylation, we first examined the status of ERK1/2 activation by western blot, and the results confirmed an increased p‐ERK1/2 level upon wild‐type PADI2 (WT) overexpression in a dose‐dependent manner (Figure 3J; Figure S4C, Supporting Information), while not by mutant PADI2 overexpression (Figure 3J; Figure S4D, Supporting Information). We also performed immunofluorescence analysis in ISI cells transfected with either WT or Mut PADI2 and then compared the status of ERK1/2 activation in these cells. Exogenous expression of Flag‐tagged PADI2 (WT) increased ERK1/2 phosphorylation above the basal level, compared to the mutant PADI2 (Figure 3K). Collectively, these results support the hypothesis that phosphorylation of ERK1/2 by MEK1 is dependent on the enzymatic activity of PADI2.

**Figure 4:**
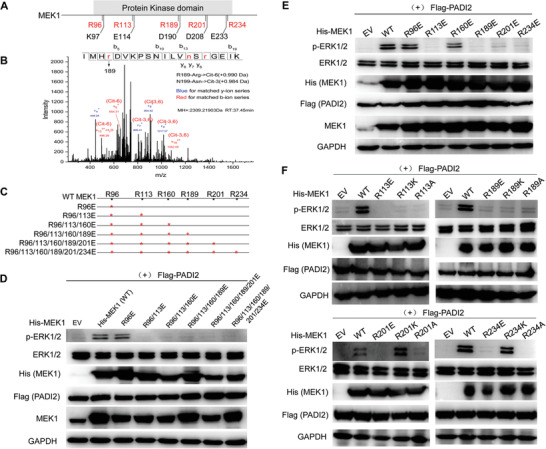
PADI2 Targets MEK1 specifically at Arg113/189 for citrullination and induces ERK1/2 activation. A) Schematic structures showing arginine sites 96/113/189/201/234 adjacent to the functionally important residues K97, E114, D190, D208, and E233 within MEK1. B) MS/MS analysis identifies citrullination of MEK1 at R189. Plot shows the fragmentation spectra of the LysC peptide IMHrDVKPSNILVnSrGEIK. C) Schematic illustration of R/E mutation of MEK1. Red stars indicating the corresponding mutant arginine sites. D) Western blot analysis of p‐ERK1/2 in ISI cells overexpressing a series of R/E mutants within MEK1, or E) individual R/E mutant, together with Flag‐PADI2. EV: empty vector control. F) Western blot analysis of p‐ERK1/2 in ISI cells overexpressing MEK1 R/E, R/K, or R/A mutant at R113, R189, R201, R234, together with Flag‐PADI2.

The activation of MEK1 is achieved by the dual phosphorylation of Ser218 and Ser222, which mediates the phosphorylation of tyrosine and threonine in ERK1/2. To maintain this catalytic activity, MEK1 contains several functionally important residues, including an invariant *β*3‐strand lysine (K97) and a conserved glutamate (E114) near the center of the *α*‐C helix to form salt bridges; histidine (H188), arginine (R189), aspartate (D190), and K192 to constitute the primary structure of the catalytic loop; as well as D208 and E233 to compose the activation segment.^[^ ^28^ ^]^ Interestingly, arginine sites 96/113/189/201/234 are just adjacent to these residues (**Figure** **4**A). Given that citrullination of substrate protein catalyzed by PADIs can regulate protein structure and function,^[^ ^14^, ^17^ ^]^ it is interesting to find out whether PADI2 could target these arginine sites and thus affect the catalytic enzymatic activity of MEK1. To test this hypothesis, we first map the sites of citrullination in MEK1. His‐tagged MEK1 was purified and treated with PADI2. The protein band corresponding to the mass of the citrullinated MEK1 was then excised from the gel and evaluated by mass spectroscopic (MS) analysis (Figure S5A, Supporting Information, insert). LC‐MS/MS analysis revealed that PADI2 preferentially citrullinates the arginine (R) residues 189 (Figure 4B), and R47, R49, R201, and R349 were also identified throughout the protein (Figure S5A–D, Supporting Information). To further confirm the role of these R96/113/189/201/234 residues on MEK1 kinase activity, we then generated R→E mutants of MEK1 on R sites as indicated in red stars (Figure 4C) and transfected them into HEK293 cells to test their activity in the presence of PADI2. Results showed that overexpression of WT MEK1 greatly increased ERK1/2 phosphorylation levels (Figure 4D), and substitution of R96 with E96 on MEK1 did not alter MEK1 kinase activity (lane 3), thus excluding the effect of R96 residue for MEK1 activity. However, double mutation of R96/113E or more mutation on R160/189/201/234E abolished the ERK1/2 phosphorylation catalyzed by MEK1 (lanes 4–8), suggesting that R113 or R160/189/201/234 may be pivotal sites for MEK1 kinase activity. Of note, in the absence of PADI2, neither WT nor any mutant form of MEK1 could keep the MEK1 kinase activity (Figure S6A, Supporting Information), raising a possibility that PADI2 may target R113 or R160/189/201/234 for citrullination. The blots were probed with anti‐His antibody to confirm the equal exogenous MEK1 expression, and total ERK1/2 levels were also evaluated with anti‐ERK1/2 antibody to normalize for protein loading.

To confirm whether single R113, R160, R189, R201, and R234 mutant could bring such effect on MEK1 kinase activity as tested above, we then generated single R→E mutant of MEK1, and co‐transfected it in the presence or absence of PADI2 (Figure 4E; Figure S6B, Supporting Information). Results showed that single R113K, R189K, R201K, or R234K mutant could inhibit ERK1/2 phosphorylation when co‐transfected with PADI2, while R96K or R160K mutant did not, suggesting that R113, R189, R201, and R234 residues are potential PADI2 targets on MEK1 (Figure 4E). Given that citrullination neutralizes positively charged arginine residues, we then generated R→A and R→K mutants for R113, R189, R201, and R234, separately, to exclude the possibility that positive charges of these residues play the roles. Note: R→K mutation does not change the positive charge. When co‐transfected with PADI2, both R113 and R189 mutants inhibited MEK1 kinase activity (Figure 4F, top panel). However, R→K mutation on R201 and R234 residues did not affect ERK1/2 activation, though R→E and R→A mutants still had the inhibitory effect (Figure 4F, bottom panel). These results suggest that the positive charges on R201 and R234 residues are critical for maintaining MEK1 kinase activity. Instead, both R113 and R189 residues are citrulline‐dependent, as R→K, R→E, and R→A mutants all decreased the p‐ERK1/2 levels, further highlighting that R113 and R189 are the target sites for PADI2 to citrullinate on MEK1, which affects MEK1 kinase activity.

To further test whether ERK1/2 activation by MEK1 is PADI2‐catalyzed R113/R189 citrullination dependent, we overexpressed Kras in both PADI2 WT and PADI2^−/−^ ISI cells, and found that p‐MEK1 can be activated under each condition. However, Kras overexpression in PADI2^−/−^ ISI cells only slightly activated p‐ERK1/2 (Figure S7A, Supporting Information, compare lane 4 with 3), while Kras in PADI2 WT cells dramatically enhanced p‐ERK1/2 (lane 2), suggesting that ERK1/2 phosphorylation by Ras/Raf/MEK1 activation is probably PADI2‐independent. In the presence of PADI2, PADI2‐catalyzed MEK1 citrullination may enhance ERK1/2 activation. To further confirm this hypothesis, we also rescued either WT PADI2 or the enzymatically inactive PADI2 in PADI2^−/−^ ISI cells (Figure S7B, Supporting Information). Rescue of WT PADI2 totally restored the p‐ERK1/2 to that in the shRNA control cells (lane 6), while the mutant PADI2 did not (lane 7). Correspondingly, both transwell invasion assay (Figure S7C,D, Supporting Information) and spheroid growth assay (Figure S7E,F, Supporting Information) demonstrated that Kras overexpression in PADI2^−/−^ ISI cells increased cell invasion and the growth of ISI derived spheroids, and rescue of WT PADI2 expression could restore the cell invasion ability and spheroid growth to that of shRNA control cells. In addition to activating MEK1 with Kras overexpression, we also inhibited Ras or Raf with Salirasib or Sorafenib, separately, to inactivate p‐MEK1 in HEK293 cells (Figure S7G, Supporting Information), and then tested whether PADI2 can rescue p‐ERK1/2. Results showed that MEK1 inactivation obviously inhibited p‐ERK1/2 (lanes 4–9). Importantly, overexpression of WT PADI2 partly restored ERK1/2 activation (compare lanes 5, 8 with lanes 4, 7), while mut PADI2 did not, suggesting that PADI2‐catalyzed MEK1 citrullination may help restore the conformation of MEK1 to its active form. Furthermore, we co‐expressed MEK1 (WT or R113/189 mutants) and Flag‐PADI2 (WT or C647S mutant) in HEK 293 cells in the absence or presence of Kras overexpression (Figure S8, Supporting Information). Though Kras overexpression can activate MEK1 and this activation slightly activated p‐ERK1/2 (compare lanes 3–5, 7–9, 11–13 with lane 1), PADI2 WT overexpression failed to further promote the phosphorylation of ERK1/2 when MEK1 R113/189 mutants were overexpressed (compare lane 9 with lane 8; lane 13 with lane 12), compare to that of WT MEK1 overexpression (compare lane 5 with lane 4). Together, these results suggest that ERK1/2 activation by p‐MEK1 may not be PADI2‐dependent, but the presence of PADI2 and PADI2‐catalyzed MEK1 citrullination at R113/189 may facilitate MEK1 binding to ERK1/2, or help maintain the active MEK1 site properly structured, thus enhances MEK1 activity on ERK1/2.

### 2.5 Overexpression of MEK1 R113E or R189E Mutant in EC Cells Leads to Decreased Cell Proliferation and ERK1/2 Phosphorylation

**Figure 5:**
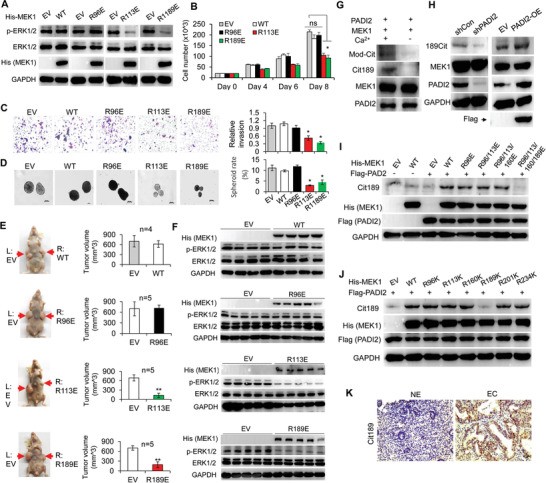
Overexpression of MEK1 R113E or R189E mutant in EC cells leads to decreased cell proliferation and ERK1/2 phosphorylation. A) Western blot analysis of p‐ERK1/2 in ISI cells overexpressing MEK1 WT, R/E mutant, or empty vector (EV) control. GAPDH served as loading control. B) Cell number counting analysis of ISI cells overexpressing MEK1 WT, R/E mutant, or EV control under light microscope. C) Representative images (left) and quantification (right) for transwell assay upon stable overexpression of MEK1 WT, R/E mutant, or EV control. D) Representative images of ISI derived spheroids (diameter greater than 100 µm) cultured at 10% FBS in concave ultra‐low attachment plates (left) and quantification (right) upon stable overexpression of MEK1 WT, R/E mutant, or EV control. E) Left: Reprehensive nude mice injected with ISI cells stably overexpressing EV control on the left flank and MEK1 WT or R/E mutant on the right flank at the experimental end point. Right: The volume of tumors for each group in nude mice was quantified. F) Protein levels of His‐MEK1, p‐ERK1/2, ERK1/2, and PADI2 in tumor lysates. GAPDH served as loading control. G) Citrullination of MEK1 by PADI2 in vitro. The reactions were assessed by western blot using anti‐Mod‐Cit, anti‐MEK1 Cit189, anti‐PADI2, and anti‐MEK1 antibody. H) Western blot analysis of Cit189 of MEK1, MEK1, PADI2 in ISI cells upon PADI2 knockdown (left), or Flag‐tagged PADI2 overexpression (right). GAPDH served as loading control. I) Western blot analysis of Cit189 of MEK1 in ISI cells overexpressed a series of MEK1 R/E mutants, or J) individual R/E mutant, together with Flag‐PADI2. K) Immunohistochemistry analysis of Cit189 in representative sections from EC patients and normal endometrium section, original magnification, × 40. Results are presented as mean ± SEM, *n* = 3. *p < 0.05, **p < 0.01.

We next generated the stable ISI cells overexpressing His‐tagged MEK1 WT, or the corresponding R96E, R113E, or R189E mutant (**Figure** **5**A). Both R113E and R189E mutants, but not R96E mutant, inhibited ERK1/2 phosphorylation, compared to the empty vector control (EV) overexpression (Figure 5A). The anti‐His antibody was used to confirm the equal exogenous MEK1 expression between the WT and the three‐individual mutants in cells. Correspondingly, the following cell proliferation assay (Figure 5B) and transwell invasion assay (Figure 5C) showed that stable overexpression of MEK1 R113E or R189E mutant suppressed ISI cell proliferation and invasion compared to the EV, while R96E mutant did not. To further test if PADI2‐catalyzed MEK1 R113/R189 citrullination modulate tumor cell viability and spheroid growth was monitored (Figure 5D). The MEK1 R113E or R189E mutant substantially decreased the growth of ISI derived spheroids when cultured in the presence of 10% FBS, indicating that MEK1 R113 or R189 citrullination is exclusively essential for tumor cell proliferation. Additionally, we performed xenograft experiments in which nude mice were injected with ISI control cells (EV, on the left flank) and cells overexpressing MEK1 WT, R96E, R113E, or R189E mutant (on the right flank), and the mice were monitored for tumor growth over 30 days (Figure 5E, left). The volumes of tumors derived from R113E and R189E cells were significantly reduced compared to those of tumors formed by control cells, while tumors derived from WT or R96E cells showed similar tumor volume to that of the control cells (Figures 5E right, and Figure S9A, Supporting Information). As expected, the ERK1/2 phosphorylation from all the R113E tumor tissues, or the R189E tumor tissues was obviously decreased compared to that of tumors inoculated with the control cells, whereas there was no difference of ERK1/2 activation in WT and R96E overexpression tumors compared to the controls (Figures 5F). Together, these results further confirmed that MEK1 kinase activity relies more on R113 and R189, but not R96 residue.

To examine the in vivo citrullination of MEK1 at the 189th arginine residue, we raised an antibody that could recognize citrullinated MEK1 using a peptide corresponding to amino acids 181 to192 (Figure S9B, Supporting Information). Note: Due to the hydroxy immuno‐peptide, there was hardly a suitable peptide for designing Cit113 antigen. Antibody titers and specificity were verified by dot blotting using synthetic citrullinated peptide (Cit189: REKHKIMH_Cit_DVK) and noncitrullinated peptide mimicking modified MEK1 (Arg189: REKHKIMH_R_DVK). The anti‐Cit189 antibody clearly indicated the citrullination of MEK1 with the same molecular weight as that of anti‐MC (45 kDa) from the purified MEK1 treated with PADI2, and was not reactive with proteins without Ca^2+^ treatment (Figure 5G). Furthermore, this antibody also readily picked up the signal from the ISI control cells, while this signal decreased upon PADI2 depletion or inhibiting PADI2 with BB‐Cla or increased along with PADI2 overexpression (Figure 5H; Figure S9C, Supporting Information), suggesting that the antibody would recognize the citrullination of R189 in a PADI2‐dependent manner. To exclude the possibility that this antibody would recognize other arginine sites in MEK1, we tested this antibody in HEK293 cells overexpressing MEK1 WT or mutants in the presence of PADI2. Results showed that mutation of R96E, R96/113E, R96/113/160E, or the single R96K, R113K, R160K, R201K, R234K did not affect the ability of anti‐Cit189 antibody to recognize R189 citrullination (Figure 5I,J). However, mutation of R189 nearly completely abolished the signal (Figure 5I,J; lane 8 and lane 6, respectively), further confirming the specificity of the antibody. We then tested the citrulline levels of R189 of MEK1 in clinical endometrial tumor tissues using immunohistochemistry, and found that Cit189 staining was strongly positive in endometrial tumor sections, while less positive or nearly negative in the normal endometrium sections (Figure 5K). These findings strongly implied that PADI2 catalyzing the citrullination of MEK1 at R189 (possibly R113) in EC cells may facilitate MEK1 kinase activity on ERK1/2 phosphorylation, thus inducing the expression of IGF2BP1 and promoting EC tumorigenesis.

### 2.6 PADI2/MEK1/ERK/IGF2BP1 Axis Maintains the Stability of SOX2 Transcript in EC Cells

**Figure 6:**
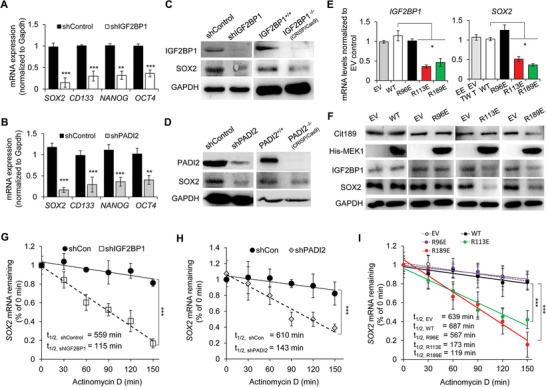
PADI2/MEK1/ERK/IGF2BP1 axis maintains the stability of *SOX2* transcript in EC cells. A) qRT‐PCR analysis of *SOX2, CD133, NANOG*, and *OCT4* levels in IGF2BP1 stable knockdown and control ISI cells, or B) stable PADI2 knockdown and control ISI cells. C) Western blot analysis of IGF2BP1 and SOX2 expression in IGF2BP1 KD or CRISPR/Cas9‐mediated IGF2BP1^−/−^ ISI cells, and D) PADI2 KD or CRISPR/Cas9‐mediated PADI2^−/−^ ISI cells, compared to the corresponding controls. E) qRT‐PCR analysis of *IGF2BP1* (left) and *SOX2* (right) in ISI cells overexpressing MEK1 WT, R/E mutant, or empty vector (EV) control. F) Western blot analysis of Cit189, His‐MEK1, IGF2BP1, and SOX2 in ISI cells overexpressing MEK1 WT, R/E mutant, or empty vector (EV) control. GAPDH served as loading control. G) The decay of the *SOX2* mRNA was monitored by qRT‐qPCR in IGF2BP1 stable knockdown and control ISI cells, H) in PADI2 stable knockdown and control ISI cells, I) in ISI cells overexpressing MEK1 WT, R/E mutant, or EV control, treated with actinomycin D for the indicated time. The reduction of the *SOX2* mRNA half‐life by IGF2BP1 knockdown is indicated in the graph. Results are presented as mean ± SEM, *n* = 3. *p < 0.05, ***p < 0.001.

We next explored the molecular mechanism by which PADI2/MEK1/ERK/IGF2BP1 axis regulates the endometrial carcinogenesis. It has been reported that poorly differentiated tumors express high levels of stemness‐related factors, including sex‐determining region Y‐box 2 (SOX2), octamer‐binding transcription factor (OCT4), CD133, and NANOG.^[^ ^30^ ^]^ Meanwhile, accumulated studies have indicated that the upregulated IGF2BP1 in aggressive cancers is associated with stabilizing mRNAs of numerous oncogenes and pluripotency factors, thus promoting cancer cell survival and enhancing a tumor‐initiating cell phenotype.^[^ ^31^, ^32^, ^33^ ^]^ We therefore wondered where IGF2BP1 may also affect the expression of these oncogenic factors in EC cells. As shown in **Figure** **6**A, depletion of IGF2BP1 in ISI cells decreased the mRNA levels of *SOX2*, *CD133*, *NANOG*, and *OCT4*, with *SOX2* being the most significant one. Given that IGF2BP1 is the downstream factor in PADI2/MEK1/ERK signaling pathway, we expected to observe the similar expression pattern of these factors upon PADI2 depletion. As shown in Figure 6B, *SOX2* mRNA level exhibited a seven‐ to eightfold of decrease in PADI2‐depleted cells, compared to the control cells. We also showed that SOX2 expression was inhibited at the protein level in IGF2BP1 or PADI2 knockdown ISI cells, as well as in CRISPR/Cas9‐mediated IGF2BP1 or PADI2 depleted ISI cells (Figure 6C,D; Figure S10A, Supporting Information). These results promoted us to focus on SOX2 as a potential target for IGF2BP1 in EC cells. Given the fact that citrullination of MEK1 R113/189 catalyzed by PADI2 may facilitate ERK1/2 activation and IGF2BP1 expression in EC cells (Figure 5), we presumed that MEK1 citrullination at the 113th and 189th arginine residues would also affect SOX2 expression. To test this hypothesis, we detected the expression of IGF2BP1 and SOX2 in ISI control cells and cells overexpressing MEK1 WT, R96E, R113E, or R189E mutant. Results showed that overexpression of MEK1 R113E or R189E mutant suppressed protein and mRNA levels of both IGF2BP1 and SOX2, while MEK1 WT or R96E mutant did not (Figure 6E,F), suggesting that SOX2 expression is also dependent on MEK1 R113/189 citrullination. Of note, ISI cells overexpressing MEK1 R189E mutant weaken the citrullination of MEK1 R189, using the anti‐MEK1 Cit189 antibody, while other mutants on R96E and R113E did not affect the ability of this antibody to recognize R189 citrullination in MEK1 (Figure 6F), further confirming the antibody specificity. Together, these results indicated that SOX2 regulation is likely to rely on PADI2‐mediated ERK1/2 activation, and the consequent enhancement of IGF2BP1 expression in the EC cells.

As an oncogenic transcriptional factor, SOX2 has been upregulated in several types of human cancers including EC and facilitates tumor initiation and progression.^[^ ^34^, ^35^ ^]^ Currently, it is not clear how IGF2BP1 regulates SOX2 expression. Previous studies have indicated that IGF2BP1 associates with other RNA stabilizers to promote the stability of their mRNA targets and control target mRNA fate.^[^ ^36^, ^37^ ^]^ A recent study has demonstrated that methylated *SOX2* transcripts were directly recognized by the RNA N^6^‐methyladenosine (m^6^A) “reader”, IGF2BP2, which maintained the stability of the transcripts to prevent *SOX2* mRNA degradation and naturally increased its expression in colorectal carcinoma.^[^ ^38^ ^]^ Given IGF2BP1 has been shown to stabilize multiple target transcripts and give rise to enhanced oncogene expression,^[^ ^31^, ^36^, ^39^ ^]^ and our results showing that inhibiting or depletion of IGF2BP1 led to a decreased SOX2 expression, we wondered whether IGF2BP1 could also help maintain the stability of *SOX2* transcripts in EC cells. To test this hypothesis, we then assessed *SOX2* mRNA decay rate in IGF2BP‐depleted EC cells and the corresponding control cells. Cells were treated with actinomycin D to inhibit transcription. The *SOX2* mRNA expression was initially decreased and its half‐life was consistently and significantly shortened in IGF2BP1‐depleted ISI cells (Figure 6G). Correspondingly, silencing PADI2 also failed to stabilize *SOX2* transcripts (Figure 6H; Figure S10B, Supporting Information). Consistent with the downregulation of *SOX2* expression in ISI cells overexpressing MEK1, R113E, or R189E mutant (Figure 5E,F), overexpression of MEK1, R113E, or R189E mutant enhanced decay of the *SOX2* mRNA, compared to MEK1 WT or R96E overexpression ISI cells (Figure 6I), suggesting that citrullination of MEK1 R113/189 catalyzed by PADI2 is a critical regulator in *SOX2* stability maintenance. From these results, we concluded that IGF2BP1 maintains the stability of *SOX2* transcript in EC cells, which is also dependent on PADI2/MEK1/ERK signaling.

### 2.7 IGF2BP1 Enhances *SOX2* mRNA Stability via an m^6^A‐dependent Manner

**Figure 7:**
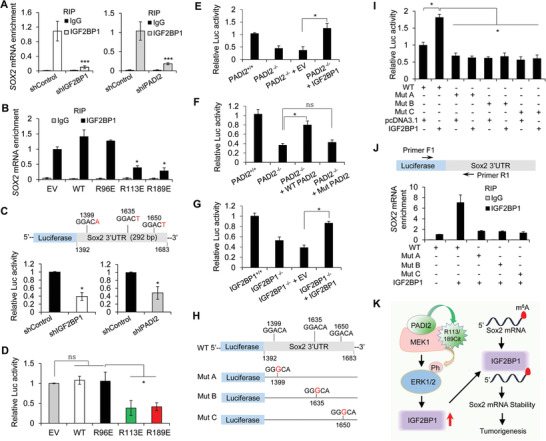
IGF2BP1 enhances *SOX2* mRNA stability via an m^6^A‐dependent manner. A) RIP‐qPCR analysis of the enrichment of *SOX2* in IGF2BP1 stable knockdown and control ISI cells (left), in PADI2 stable knockdown and control ISI cells (right), B) in ISI cells overexpression of MEK1 WT, R/E mutant, or EV control. Differential enrichment was normalized to that of shControl or EV condition and plotted as ratio. IgG used as negative control. C) Top: Schematic representation showing luciferase reporter construct comprising indicated region of the *SOX2*‐3′UTR (292 bp) including three m^6^A consensus sites “GGACH” with respective mRNA coordinates. Bottom: Luciferase reporter analysis of *SOX2*‐3′UTR‐driven firefly luciferase reporter in IGF2BP1‐deleted (bottom, left), and in PADI2‐depleted (bottom, right) compared to the respective parental control cells. D) Luciferase reporter assays using *SOX2*‐3′UTR‐driven firefly luciferase reporter construct in ISI cells overexpressing MEK1 WT, R/E mutant, or EV control. Reporter activity was determined relative to cells transfected with EV control. E) Luciferase reporter analysis of *SOX2*‐3′UTR‐driven firefly luciferase reporter constructs in CRISPR/Cas9‐mediated PADI2 KO ISI cell rescued with IGF2BP1 overexpression, F) WT or Mut PADI2 overexpression, and G) in CRISPR/Cas9‐mediated IGF2BP1 KO ISI cell rescued with IGF2BP1 overexpression. H) Schematic representation showing the reporters with point mutation of A to G (by red letters) at three m^6^A consensus sites within *SOX2*‐3′UTR. I) Luciferase reporter analysis of *SOX2*‐3′UTR‐driven firefly luciferase reporter constructs with individual mutants in HEK293 cells with ectopically expressed IGF2BP1. J) Top: Schematic representation of *SOX2*‐3′UTR‐driven firefly luciferase reporter and respective primer pairs F1/R1, which amplifies exogenous 3′UTR reporter‐derived *SOX2*. Bottom: RIP‐qPCR analysis of the enrichment of exogenous Luc‐3′UTR‐derived *SOX2* RNA in ISI cells co‐transfected with individual mutants shown in (E) and IGF2BP1. K) Proposed model of the function of MEK1 citrullination at R113 and R189 in regulation of ERK1/2 activation, and the *SOX2* mRNA stability. PADI2 catalyzed R113/189 to Cit113/189 in MEK1, facilitates MEK1 kinase activity on ERK1/2 phosphorylation and activates IGF2BP1 expression, helping maintain *SOX2* mRNA stability and SOX2 expression, and therefore supporting the malignant state of EC.

Recent studies have revealed an enrichment of three non‐redundant m^6^A sites (GG**A**CH) around 3′UTR of *SOX2* gene, and m^6^A modification on these sites resulted in *SOX2* mRNA stabilization with the subsequent increase in SOX2 protein levels.^[^ ^40^, ^41^ ^]^ As m^6^A “readers”, IGF2BPs have been shown to play specific roles in controlling the fate of the methylate mRNA by targeting to these m^6^A sites.^[^ ^36^, ^42^ ^]^ We therefore first performed RNA immune precipitation (RIP) assay to calculate the binding affinity of IGF2BP1 to *SOX2* mRNA in ISI cells. RIP‐qPCR using anti‐IGF2BP1 antibody brought down *SOX2* mRNA efficiently, but the binding was diminished upon IGF2BP1 depletion (**Figure** **7**A, left), thus validating the direct interaction between the IGF2BP1 and *SOX2* mRNA. Furthermore, this specific binding was impaired in ISI cells depletion of PADI2 compared to that in the parental control cells (Figure 7A, right; Figure S10C, Supporting Information), or in cells overexpressing MEK1, R113E, or R189E mutant relative to EV or WT MEK1 overexpression (Figure 7B), suggesting that PADI2‐catalyzed MEK1, R113/189 citrullination is also required for IGF2BP1 binding to *SOX2*. Then, we generated a luciferase reporter construct harboring 292‐bp fragment of *SOX2*‐3′UTR, which contains three m^6^A sites (shown in the upper panel in Figure 7C) that are non‐redundant in maintaining the *SOX2* mRNA stabilization.^[^ ^41^ ^]^ After transfection of luciferase‐*SOX2* 3’UTR construct into IGF2BP1 or PADI2 KD ISI cells and their corresponding control cells for 48 h, cells were collected and luciferase reporter assay was performed. Silencing IGF2BP1 or PADI2 inhibited the luciferase activity (Figure 7C; Figure S10D, Supporting Information), indicating a critical role of IGF2BP1 in regulating luciferase activity by targeting to *SOX2*‐3’UTR. We also transfected this reporter into ISI cells overexpressing MEK1 WT, R96E, R113E, or R189E mutant. The luciferase activity was largely impaired in cells with R113E or R189E mutant overexpression, compared to the control cells, or cells with MEK1 WT or R96E overexpression (Figure 7D). To further confirm our hypothesis that IGF2BP1 is the downstream target of PADI2/MEK1/ERK signaling and that PADI2‐catalyzed MEK1 citrullination is required for IGF2BP1‐mediated *SOX2* expression, we then performed the luciferase reporter assay again in CRISPR/Cas9‐mediated PADI2^−/−^ ISI cells rescued with IGF2BP1 expression. As shown in Figure 7E, the decreased luciferase activity due to the depletion of PADI2 was completely restored by ectopic IGF2BP1 overexpression. Furthermore, PADI2 enzyme activity was proved to be essential since only the forced expression of WT PADI2 can rescue the decreased luciferase activity, but not the enzymatically inactive mutant PADI2 (Figure 7F). As a control, rescued expression of IGF2BP1 in IGF2BP1^−/−^ ISI cells also restored the luciferase activity (Figure 7G). These results suggested that IGF2BP1 may target these three m^6^A sites in *SOX2*‐3′UTR and contribute to *SOX2* mRNA stability in EC cells, and PADI2‐catalyzed MEK1 arginine 113/189 citrullination is required for IGF2BP1 binding and regulating *SOX2* mRNA stability.

Next, we expected to test whether the presence of all three m^6^A sites are required for IGF2BP1 binding to *SOX2*‐3′UTR. We generated point mutation, separately, in the three m^6^A sites (“A” converted to “G”) of *SOX2*‐3′UTR and then inserted the sequence into the luciferase reporter construct (Figure 7H; Mut A, Mut B, and Mut C) as shown previously.^[^ ^41^ ^]^ Overexpression of IGF2BP1 induced a significant increase in luciferase activity from WT reporter, compared to the empty vector control overexpression (Figure 7I; compare bar 2 with 1), but such an increase was impaired by mutation in any one of the m^6^A sites of the reporter (Figure 7I; compare bars 3–8 with 2). To further confirm the three m^6^A sites on the *SOX2*‐3′UTR are non‐redundant in IGF2BP1 binding, we performed RIP assay again with anti‐HA (IGF2BP1) antibody to detect the pulldown efficiency of *SOX2*‐3′UTR reporter derived transcript using primer set F1/R1 which amplifies fusion region of luciferase‐*SOX2*‐3′UTR. Results demonstrated a strong binding of exogenous overexpression of IGF2BP1 with WT *SOX2*‐3′UTR reporter‐derived transcript (Figure 7J; compare bar 4 with 2), but not with the reporter with mutation in any one of the m^6^A sites on *SOX2*‐3′UTR (Figure 7J; compare bars 6, 8, 10 with 2). As an internal control, we also tested the pulldown efficiency of endogenous *SOX2* transcript by IGF2BP1 using primer pair F2/R2 that amplifies exon region of *SOX2*. Compared to the empty vector overexpression, ectopic IGF2BP1 induced an increase in interaction with endogenous *SOX2* transcript when co‐transfected with either WT or mutated *SOX2* 3′UTR construct (Figure S11, Supporting Information;compare bars 4, 6, 8, 10 with 2). Taken together, our data suggested that the three non‐redundant m^6^A sites on *SOX2*‐3′UTR are indispensable for IGF2BP1 binding to *SOX2* 3′UTR, thus regulating IGF2BP1‐mediated *SOX2* stability in EC cells.

## 3 Discussion

The expression of PADI2 in regulation of tumorigenesis seems to be cell‐type and tumor microenvironment‐dependent. For example, PADI2 expression is upregulated in several digestive system cancers,^[^ ^43^ ^]^ while downregulated in colon cancer.^[^ ^44^ ^]^ PADI2 also displays a controversial expression pattern in highly heterogeneous breast cancer.^[^ ^12^ ^]^ These findings testify to the significance of PADI2 in tumor biology. However, no previous studies have experimentally investigated the tumorigenic potential of PADI2 in EC. Here we demonstrate that PADI2 expression is upregulated in EC patients, suggesting that PADI2 may be involved in regulation of EC progression. To test this hypothesis, we then investigated the effect of PADI2 depletion on EC cell malignant behavior in two well‐established EC cell lines both in vitro and in vivo and found that knockdown of PADI2 suppresses the ability of EC cells to proliferate, migrate, invade, and metastasize. From these data, we believe that PADI2 drives EC tumorigenesis.

A key finding in the present study is that PADI2 directly citrullinates MEK1 and facilitates MEK1 kinase activity in EC cells. Numerous studies are convinced that PADI2‐catalyzed protein citrullination largely reflects the function of PADI2 in cancers.^[^ ^12^, ^14^, ^43^, ^45^ ^]^ For example, PADI2 citrullinating R1810 at the C‐terminal domain of RNA polymerase II (RNAP2) facilitates interaction of RNAP2 with p‐TEFb complex and subsequently regulates transcription and proliferation of breast cancer cells.^[^ ^46^ ^]^ Our previous study showed that PADI2 specifically citrullinates histone H3R26 citrullination that leads to chromatin decondensation and transcriptional activation in human breast cancer cells,^[^ ^47^ ^]^ and the following study from Han group further demonstrated that citrullination of H3R26 by PADI2 activates androgen receptor signaling and promotes prostate cancer progression.^[^ ^14^ ^]^ These studies indicate a tumor‐promoting effect of citrullination in multiple types of cancers. Consistent with the citrullination activity in these reports, we identified citrullination of MEK1 by PADI2 in EC cells, specifically at R113 and R189 residues within MEK1 kinase catalytic domain. Inhibition of citrullination by BB‐CLA, PAD2 gene silencing, or mutation of R113/R189 equally reduced MEK1 kinase activity. Further, this modification is coupled with the MEK1 kinase activity to activate ERK1/2 phosphorylation, leading to the upregulated expression of IGF2BP1 and its downstream target SOX2 and thus driving EC tumor progress. Given that citrullination neutralizes positively charged arginine residues and alters protein structure, and that R113 and R189 are just next to conserved amino acid residue signatures that constitute the salt bridge and catalytic loop within MEK1 kinase domain,^[^ ^28^ ^]^ it is conceivable that PADI2‐mediated MEK1 citrullination facilitates MEK1‐catalyzed phosphorylation of ERK1/2 by allowing this kinase closer access to its substrate ERK1/2. It is also possible that citrullination of MEK1 may help maintain the active MEK1 site properly structured, thus enhances ERK1/2 activation by MEK1. Of note, we cannot exclude the possibility that loss of function of the R113/189 mutants is due to destabilization of MEK1, in a way that is not dependent on PADI2. Though the R113/189 mutants do not seem to affect the expression levels of MEK1 protein, we do not know yet whether these mutants still maintain MEK1 properly structured. This is worthy of further investigation. MEK1/2 plays crucial roles in tumorigenesis. In particular, their unique structures, narrow substrate specificity, and minimum prevalence of mutation rates in human tumors attract growing interests among pharmacological researchers.^[^ ^48^ ^]^ However, the clinical benefits of MEK inhibitors are discounted because of the frequently occurring acquired resistance as a result of cancer heterogeneity and genomic instability.^[^ ^49^ ^]^ It is now clear that combination of different inhibitors of the same target, or drugs for different targets within the same pathway, can expand drug efficacy compared with single inhibitor.^[^ ^7^ ^]^ Actually, our previous study has demonstrated a synergistic and effective drug regimen of PADs inhibitor and docetaxel on tamoxifen‐resistant breast cancer cells.^[^ ^16^ ^]^ Therefore, we provide here an important new line of evidence demonstrating that inhibiting conversion of MEK1 R189/R113 to citrulline by PADI2 inhibitor compromises MEK1 kinase activity in cancer cells, which may offer the promise of a novel therapy with combination of PADI2 inhibitors and MEK1 inhibitors on EC malignancies.

Our finding that IGF2BP1 expression is downregulated upon PADI2 depletion, inhibiting MEK1 citrullination, or mutation of MEK1 R113/R189, suggests that IG2BP1 is controlled by PADI2‐catalyzed MEK1 citrullination. Given that ERK1/2 are specific substrates of MEK1, and that inhibiting ERK1/2 phosphorylation also decreases IGF2BP1 expression, we believe that IGF2BP1 is a downstream effector in PADI2/MEK1/ERK/IGF2BP1 pathway. However, it is not clear how p‐ERK1/2 activate IG2BP1 expression. As ERK1/2 have a wide range of substrates,^[^ ^27^ ^]^ it is possible that IGF2BP1 represents one of them. A recent study has demonstrated that IGF2BP1 is a novel p38 MAPK‐interacting protein,^[^ ^50^ ^]^ it will be interesting to further test whether ERK1/2 could also directly target IGF2BP1. Additionally, IGF2BP1 has shown to activate ERK, JNK, and p38 MAPKs, which eventually promoted the progression of hepatocellular carcinoma cells,^[^ ^51^ ^]^ therefore, we cannot exclude the possibility there is a reciprocal regulatory loop between ERK1/2 and IGF2BP1. Actually, a positive feedback regulation has been demonstrated between IGF2BP1 and *β*‐catenin,^[^ ^52^ ^]^ and IGF2BP1 and c‐Myc also regulate each other's expression in a positive feedback regulation analogous to that created by IGF2BP1 and *β*‐catenin.^[^ ^53^ ^]^ More work is required to decipher this cross‐talk and feed‐back regulation which are likely to orchestrate IGF2BP1 transcription in EC. As an oncofetal protein, IGF2BP1 is predominantly expressed in various cancers,^[^ ^20^, ^50^, ^51^, ^54^ ^]^ and promotes a mesenchymal tumor cell phenotype and drive tumor progression.^[^ ^22^, ^23^, ^33^ ^]^ Due to its conserved oncogenic role in tumors, IGF2BP1 has been proposed as a potential target to develop novel drugs to treat cancer without deleterious side effects from targeting noncancerous cells.^[^ ^50^, ^55^ ^]^ Our finding that PADI2 gene silencing, inhibition of MEK1 citrullination by BB‐CLA, mutation of R113/R189, or depletion of IGF2BP1 exhibits similar effects on inhibiting EC tumor cell proliferation and invasion supports the notion that PADI2/MEK1/ERK/IGF2BP1 pathway promotes oncogenic tumor cell properties in EC.

IGF2BP1 is a conserved RNA‐binding protein, which recognizes the consensus GG(m^6^A)C sequence and target thousands of mRNA transcripts to modulate mRNA fate such as RNA localization, RNA stability, and translational control.^[^ ^36^, ^40^, ^52^ ^]^ We added *SOX2* mRNA as a novel IGF2BP1 target by the following new lines of evidence. First, we found that SOX2 expression is diminished upon IGF2BP1 depletion. Second, the depletion of IGF2BP1 led to enhanced decay of the *SOX2* mRNA, and PADI2‐catalyzed MEK1 citrullination at R113/R189 is also required to mediate IGF2BP1 activity on *SOX2* stability regulation. Third, IGF2BP1‐RIP and luciferase report analyses demonstrated that IGF2BP1 could bind to the non‐redundant three m^6^A sites in *SOX2*‐3′UTR. Our finding is in line with other reports showing that IGF2BP1 can inhibit mRNA decay and promote translation by binding to m^6^A modification of several target transcripts,^[^ ^22^, ^33^, ^53^, ^56^ ^]^ thus confirming IGF2BP1 as a m^6^A‐reader for *SOX2*. It is worth noting that IGF2BP2, the other IGF2BP family member, has also been shown to bind to *SOX2* and prevent *SOX2* mRNA degradation via an m^6^A‐dependent manner in colorectal carcinoma, to preserve the tumor stemness phenotype. However, instead of binding to *SOX2*‐3’UTR, IGF2BP2 directly bind to the specific m^6^A sites in *SOX2* CDS regions.^[^ ^38^ ^]^ These different binding patterns probably occur in a cell‐type and context‐specific nature. Moreover, multiple studies have shown that overexpression of SOX2 is contributing to cancer pathogenesis.^[^ ^57^ ^]^ In endometrial cancer, SOX2 expression is correlated with unfavorable histological grade and poor prognosis in patients, suggesting that SOX2 may be a critical factor for the proliferation of EC cells.^[^ ^35^ ^]^ These data nicely demonstrated that the dysregulation of IGF2BP1 by PADI2/MEK1/ERK signaling axis may result in abnormal accumulation of oncogenic SOX2 expression, and therefore supports the malignant state of EC (Figure 7K).

In all, this is the first study to establish a link between protein citrullination and mRNA stability regulation involved in the progression of cancer cells. These findings may help to improve our understanding of how PADI2 regulates mRNA stability via MEK1 citrullination. Thus, our finding opens the possibility that specific inhibition of citrullination at R113/R189 of MEK1 may represent a suitable drug target for EC. Most importantly, combination of specific PADI2 inhibitor with MEK1 inhibitors will probably provide a novel potential therapeutic approach in patients with EC.

## 4 Experimental Section

##### Cell Culture

Ishikawa (ISI) and ECC‐1 cells were maintained in RPMI‐1640 media (Gibco, USA) supplemented with 10% newborn calf serum (Gibco, USA) and 10% fetal bovine serum (Gibco, USA), separately. HEK293 cells were maintained in DMEM supplemented 10% fetal bovine serum, at 37 °C in a humidified 5% CO2 incubator. All lines were routinely tested for mycoplasma contamination by using a Myco‐Blue Mycoplasma Detector kit (Vazyme, China) and found to be negative.

For shRNA‐mediated PADI2 or IGF2BP1 knockdown (KD), the Mission lentiviral transduction particles containing a short hairpin RNA (shRNA) construct targeting the human PADI2 coding sequence (Sigma SHCLND‐NM_007365), or a shRNA construct targeting the human IGF2BP1 coding sequence (Sigma SHCLND‐NM_006546) were transduced into ISI and ECC‐1 cells, separately. In the control group, cells were transduced with a non‐targeting shRNA lentiviral construct (Sigma SHC002V). The shRNA sequences were summarized in Table S1, Supporting Information. Stable PADI2‐depleted or IGF2BP1‐depletedcells were selected by medium containing 1 µg mL^−1^ puromycin or 200 µg mL^−1^ zeocin.

For CRISPR/Cas9‐mediated PADI2 or IGF2BP1 knockout (KO) in ISI cells, two sgRNA (sequence summarized in Table S1, Supporting Information) were cloned into pGL3‐U6‐sgRNA vector. Cells were co‐transfected with CRISPR sgRNA plasmids and pST1374‐N‐NLS‐flag‐linker‐Cas9 plasmid. Both pGL3‐U6‐sgRNA and pST1374‐N‐NLS‐flag‐linker‐Cas9 vectors were obtained from Dr. Bin Sheng (Nanjing Medical University, Nanjing, China). Stable PADI2‐KO or IGF2BP1‐KO cells were selected by medium containing 1 µg mL^−1^ puromycin and 2µg mL^−1^ blasticidin (Invitrogen).

For cell proliferation assay, cells were seeded into 6‐cell plates at a density of 20 000 cells per well and assessed by cell counting within 8 days post cell seeding. Where indicated, BB‐Cl‐amidine was diluted in cell culture medium at the final concentration of 10, 20, 50, and 100 µm, respectively, and added to cells for indicated time before harvest. Where indicated, cells were serum starved for 12 h and subsequently stimulated with U0126 before harvest.

##### Transwell Invasion Assay

A transwell invasion assay was performed in 24‐well plates with 8 µm pore size chamber inserts (Corning, USA), according to the protocols recommended by the manufacturer. Briefly, the upper surface of the filter was coated with 100 µL of Matrigel diluted 1:9 in serum‐free 1640. Approximately 4 × 10^4^ cells were added to the upper chamber of Matrigel‐coated transwell plate (Corning) and cultured in serum‐free 1640. The lower compartment of the transwell chamber was filled with 600 µL complete media. Cells on the lower surface were then fixed with 4% paraformaldehyde, stained with 0.1% crystal violet, and photographed in three independent fields for each well under light microscope at a magnification of × 40. The relative invasion ability was calculated using image J software.

##### Wound‐Healing Assay

Cell migration was assessed using wound‐healing assay. Cells were seeded in 6‐well plates and grown to full confluence in complete media, with three parallel wells for each condition. The monolayer was scratched with a 10 µL pipette tip, and washed twice with serum‐free DMEM to remove the detached cells. The wounded areas were observed and imaged under microscope. The distances were imaged at 0, 3, 6, and 24 h after scratches, respectively. The changes in cell migration were determined by comparing the difference in wound‐healing areas at least at four fields using ImageJ (National Institute of Mental Health, MA, USA). The data from three independent experiments were used in the calculation of the final data.

##### Spheroid Growth Assay

The analysis of 3D spheroid growth was performed as previously described.^[^ ^32^ ^]^ In brief, 1000 cells as a single cell suspension per well were seeded in an ultralow attachment flat bottom 6‐well plate (Corning 3471) using FBS‐containing (10%) DMEM medium. Spheroid growth was monitored for 7 days by light microscopy, and only spheroids larger than 100 µm in diameter was counted.

##### Quantitative Real‐Time PCR

Total RNA was isolated from cells with TRIzol (Invitrogen, USA). A HiScript II qRT Super Mix (Vazyme, China) was used to synthesize the first strand of cDNA. Quantitative real‐time PCR was performed using the Hieff qPCR SYBR Green PCR Master Mix (Yeasen, China) with gene‐specific primers. The primers were listed in the Table S2, Supporting Information. All target gene transcripts were normalized to *GAPDH*, and the relative fold change in expression calculated using the 2^−ΔΔCT^ method.

##### Mutagenesis

Point mutation in the constructs was mutated using QuikChangemulti Site‐Directed Mutagenesis kit (200514, Agilent), or Mut Express II Fast Mutagenesis Kit V2 (C214, Vazyme) according to manufacturer's instructions. Mutations were confirmed by Sanger sequencing. Mutation primers are provided in Table S3, Supporting Information.

##### Western Blot

The cells were washed twice with cold PBS and then harvested for western blot. Cells were lysed in cold radioimmunoprecipitation assay (RIPA) buffer (50 mm Tris‐HCl, pH 7.4, 150 mm NaCl, 1%Triton X‐100, 1% sodium deoxycholate, 0.1% SDS) containing protease inhibitors for 30 min. The lysates were then centrifuged and the supernatants collected. Approximately 40 µg of total protein was denatured and separated by 10% SDS‐PAGE, and then transferred to a polyvinylidene fluoride membrane. The membranes were blocked with 5% non‐fat milk in Tris‐buffered saline containing 0.1% Tween‐20 (TBST) for 2 h at room temperature. The membranes were then incubated with primary antibodies overnight at 4 °C. The primary antibodies were listed in the Table S4, Supporting Information. GAPDH was used as a loading control. The membranes were washed five times with TBST and then incubated with horseradish peroxidase‐conjugated secondary antibodies for 1 h at room temperature. The signal was visualized using an Enhanced Chemiluminescence Detection Kit (Pierce Biotechnology, USA). When indicated, Ishikawa or HEK293 cells were transfected with Flag‐PADI2 (WT or C647S mutant), Flag‐Kras, and MEK1 (WT, R113E, R189E mutant) plasmids using FuGENE 6 (Roche). Where indicated, cells were treated with 50 µm Salirasib (HY‐14754, MCE) or 10 µm Sorafenib (HY‐10201, MCE).

##### Immunoprecipitation Assay

Flag‐tagged PADI2 in pcDNA3.1 (+) or control vector were transfected into HEK 293 cells together with His‐tagged MEK1 using FuGENE 6 (Roche). The whole cell lysates were immunoprecipitated with anti‐Flag M2 affinity gel (Sigma A2220). Immunoprecipitates were washed and analyzed by western blot using anti‐His and anti‐Flag antibodies as indicated. Reciprocally, cell lysates were immunoprecipitated with anti‐His antibody, followed by western blot detection with anti‐His, and anti‐Flag antibodies.

##### Immunohistochemistry

Tumor samples were collected from Department of Gynecology and Obstetrics, the Second Affiliated Hospital of Nantong University, China. Samples were collected according to patients’ written informed consent and the study was carried out according to the Institute ethics guidelines. Tissue sections were deparaffinized, rehydrated, and then incubated for 20 min in 3% hydrogen peroxide to quench endogenous peroxidase activity. Sections were then heated to retrieve the antigen in 0.01 m citrate buffer (pH 6.0) for 15 min and then blocked with 10% goat serum in PBS. Immunohistochemical analyses were performed using a Histostain Kit (Invitrogen, USA) with primary antibodies overnight at 4 °C. Sections stained were examined using a Zeiss Axio Observer microscope. Nonimmunized rabbit or mouse IgG was served as a negative control.

##### Immunofluorescence Staining

Cells were grown on glass slides in 12‐well plates. After washing with PBS, cells were fixed with 4% paraformaldehyde for 30 min, then permeabilized with 0.1% Triton X‐100 for 10 min and blocked with 5% BSA in PBS for 1 h at room temperature. Primary antibodies were added to the cells overnight at 4 °C, and Fluor 488‐conjugated goat anti‐mouse or Fluor 546‐conjugated goat anti‐rabbit secondary antibody was employed to detect fluorescence. The nuclei were stained with DAPI (Vector Laboratories, Cambridgeshire, UK). Representative images were collected with LSM 700 laser scanning confocal microscope (Carl Zeiss). When indicated, Ishikawa cells were transfected with Flag‐PADI2 and His‐MEK1 plasmids using FuGENE 6 (Roche). Forty‐eight hours later, cells were collected for immunofluorescence staining. Where indicated, Ishikawa cells were transfected with Flag‐PADI2 (WT) or enzymatically inactive mutant of PADI2 (mutation of Cys^647^ into Ser^647^, Mut), followed by immunofluorescence analysis.

##### Antibody Purification from Crude Sera

For generating site‐specific citrulline antibodies against MEK1 R189 citrulline, two rabbits were immunized with MEK1 peptides (aa 181–192) citrullinated at residues 189 (Cys + REKHKIMH_Cit_DVK) that was c‐terminally coupled to KLH. Two weeks post the initial immunization, three boost injections every 2 weeks were performed. Crude serum of the final bleed was collected for antibody purification. 5 mg of citrullinated (same as immunogen) and unmodified (Cys+REKHKIMH_R_DVK) MEK1 peptides were used to prepare antigen affinity chromatography. The crude sera of two rabbits was purified separately. The antiserum was purified by citrullinated peptide affinity column, and the cross reaction was removed by unmodified peptide affinity column. The purified antibodies were eluted in elution buffer (0.1 m glycine HCl pH 3.0), and ice‐cold neutralization buffer (1 m Tris HCl pH 8.5) was added and mixed thoroughly to neutralize the pH to 7.2.

##### Dot Blot Analysis

Citrullinated and unmodified MEK1 peptides were coated on nitrocellulose membrane and incubated at 37 °C for 2 h. After blocking in 5% non‐fat milk, the membrane was incubated with the anti‐MEK1 Cit189 antibody for 1 h at room temperature. The membranes were washed five times with TBST and then incubated with horseradish peroxidase‐conjugated rabbit IgG for 30 min at room temperature. The signals were visualized using an Enhanced Chemiluminescence Detection Kit (Pierce Biotechnology, USA).

##### PADI Enzymatic Activity Assay

MEK1 proteins were expressed and purified from pET28b‐MEK1 using Ni‐NTA Protein Purification System (Qiagen) according to manufacturer's instructions. PADI2 proteins were expressed and purified from pcDNA3.1‐Flag‐PADI2 using anti‐Flag M2 affinity gel system (Sigma). The PADI assay was performed essentially as described previously.^[^ ^29^ ^]^ The expressed MEK1 was treated with PADI2 in PADI buffer containing 50 mm Tris‐HCl, pH 7.6, 4 mm DTT, 4 mm CaCl_2_ at 37 °C for 4 h. Citrullination of MEK1 was detected using an Anti‐Citrulline (Modified) Detection Kit (Millipore 17‐347, Billerica, MA).

##### Mass Spectrometry and Identification of Citrullinated Peptides

The MS analysis was performed in Aimsmass Co. (Shanghai, China). Briefly, the PADI2‐treated MEK1 bands were excised from the gel and treated with 10 mm DTT for 45 min at 57 °C, followed by re‐acting with 15 mm iodoacetamide for 45 min in the dark. The sample was then digested with Lys‐C (VA1170, Promega) at 37 °C for 16 h. Peptide samples were then desalted and lyophilized. The sample were then resuspended with 0.1% formic acid and loaded onto an HPLC chromatography system, Fisher Easy‐nLC 1000 equipped with a C18 column. Peptides were separated by an HPLC gradient (4–18% buffer B in 182 min; and 18–90% in 13 min at a flow rate of 300nL min^−1^; buffer A = 0.1% formic acid, buffer B = 100% acetonitrile). Mass spectrometry analysis were carried out in the positive‐ion mode with full scans (350–1600 *m*/*z*) at a mass resolution of 30 000. The MS/MS spectra were searched using the software Proteome Discoverer 1.4 (Thermo). The fragment mass tolerance was set to 0.05 Da. Carboxyamidomethylation on cysteine (+57.021 Da) was set as a fixed modification, whereas citrullination of Arg (+0.9848 Da) was set as a variable modification. All MS/MS analyses indicating citrullinated peptide fragments were then manually confirmed.

##### RNA‐seq Analysis

Total RNA was isolated from PADI2 knockdown or control Ishikawa cells using miRNeasy Kit (Qiagen). A total amount of 3 µg RNA per sample was used as input material for the RNA sample preparations. Sequencing libraries were generated using NEBNext UltraTM RNA Library Prep Kit for Illumina (NEB, USA) following manufacturer's recommendations and index codes were added to attribute sequences to each sample. Finally, PCR products were purified (AMPure XP system) and library quality was assessed on the Agilent Bioanalyzer 2100 system. Each group was sequenced in duplicate. The library preparations were sequenced on an Illumina Hiseq platform and 50 bp single‐end reads were generated. Index of the reference genome was built using Bowtie v2.2.3 and clean reads were aligned to the reference genome using TopHat v2.0.12. HTSeq v0.6.1 was used to count the reads numbers mapped to each gene. And then FPKM of each gene was calculated based on the length of the gene and reads count mapped to this gene. The average gene expression values were used for the following analysis. The resulting *p*‐values were adjusted using the Benjamini and Hochberg's approach for controlling the false discovery rate. Genes with an adjusted *p*‐value < 0.01 found by DESeq were assigned as differentially expressed. Raw FASTQ files for the RNA‐seq libraries are deposited in the NCBI Sequence Read Archive (SRA) and have been assigned SRA accession: PRJNA636622.

##### Luciferase Reporter Assay

The WT 3′‐UTR of human *SOX2* mRNA was cloned and introduced between the KpnI and NheI restriction sites into the modified pGL3 luciferase reporter vector (gift from Dr. Chun Lu from Nanjing Medical University). For the mutation of the *SOX2* 3'UTR m^6^A site, a mutation was adopted from A to G. The firefly luciferase vector was used for internal control. The constructs were confirmed by sequencing. Then a mixture of luciferase reporter plasmid and IGF2BP1 plasmid or the corresponding controls were co‐transfected into HEK293 cells. Cells were harvested after 48 h of transfection. The luciferase activity was measured by the Dual‐Luciferase Reporter Assay System (E2920, Promega), normalized on equal quantity of protein measured by Bradford assay.

##### RNA Immunoprecipitation

Cells were lysed in lysis buffer (50 mm Tris‐HCl, pH 7.5, 100 mm KCl, 12 mm MgCl2, 1 mm DTT, 1% NP‐40, 1 × PI, and 200 U mL^−1^ RNase inhibitor) for 1 h on ice. After centrifugation, supernatant was collected and was mixed with 5 µg of anti‐IGF2BP1 or anti‐HA antibody overnight at 4 °C. The precleared Protein A/G agarose beads (Santa Cruz) were then added and incubated at 4 °C for another 4 h. Beads were washed three times with low‐salt solution (50 mm Tris‐HCl, pH 7.5, 100 mm KCl, 12 mm MgCl2, 1% NP‐40, and 200 U mL^−1^ RNase inhibitor), followed by two additional washes with high‐salt solution (50 mm Tris‐HCl, pH 7.5, 300 mm KCl, 12 mm MgCl2, 1mM DTT, 1% NP‐40, and 200 U mL^−1^ RNase inhibitor). Finally, they were treated with proteinase K (0.8 mg mL^−1^l) for 1 h at 37 °C. RNA was finally extracted with TRIzol.

##### RNA Stability Assay

Ishikawa cells were treated with 5 µg mL^−1^ actinomycin D (Catalog #A9415, Sigma) 24 h post cell seeding. After 30, 60, 90, 120, and 150 min incubation, cells were collected and RNA was extracted for RT‐PCR analysis. Within a given time, the decrease of the target mRNA concentration is proportional to its mRNA decay constant (*K*_decay_) and the initial concentration of mRNA. The half‐life (*t*_1∕2_) of mRNA was calculated using ln2/*K*_decay_ and GAPDH was used for normalization.

##### Xenograft Tumor Model in Nude Mice

All animal experiments were conducted according to the standard institutional guidelines of Nanjing Medical University (Nanjing, China). All female BALB/c nude (6 weeks old) mice used in this experiment were purchased from the Shanghai Laboratory Animal Center (Chinese Academy of Sciences, Shanghai, China) and maintained in special pathogen‐free environment. Nude mice were subcutaneously injected with 1 × 10^7^ PADI2 depleted or IGF2BP1 depleted Ishikawa cells on the left flanks, and the corresponding control cells on the right flanks. For evaluating the tumorigenesis potential of MEK1 mutation, 1 × 10^7^ Ishikawa cells overexpressing WT, R96E, R113E or R189E mutant were injected subcutaneously on the left flanks, and the corresponding control cells on the right flanks. Tumor diameters are measured with digital calipers, and the tumor volume in mm^3^ was calculated by the following formula: Volume = 0.5 × (width)^2^ × length. Mice were sacrificed 30 days after injection under anesthesia. The tumors were harvested and at the experimental endpoint, and the volume of tumors (mm^3^) in both flanks of each mouse were compared.

##### Statistical Analysis

All experiments were performed at least three independent biological replicates. Data are presented as mean ± SE. Statistical evaluation for data analysis was determined by Student's *t*‐test or one‐way ANOVA with *p<0.05, **p<0.01, ***p<0.001, indicating significantly different from control.

## Conflict of Interest

The authors declare no conflict of interest.

## Author Contributions

T.X., X.L., and M.Z. contributed equally to this work. X.Z. and X.L. designed the study and wrote the paper; T.X. and M.Z. contributed to the experiments, interpretation, and data analysis; Q.E., T.S., and M.Z. helped with experimental operation, and data acquisition; Z.M., Y.H., and Y.L. provided and analyzed the clinical data; P.T. edited the manuscript.

## Supporting information

Supporting Information
