# Supplementary material for: PADI2‐Catalyzed MEK1 Citrullination Activates ERK1/2 and Promotes IGF2BP1‐Mediated SOX2 mRNA Stability in Endometrial Cancer
Source: Adv Sci (Weinh). 2021 Jan 29;8(6):2002831. doi: 10.1002/advs.202002831 (PMC7967072; doi:10.1002/advs.202002831)
Supplement: Supplementary file 1 — Supporting Information [file ADVS-8-2002831-s001.pdf]

© 2020 Wiley-VCH GmbH

ADVANCED SCIENCE
Open Access

Supporting Information

for *Adv. Sci.,* DOI: 10.1002/advs.202002831

PADI2-Catalyzed MEK1 Citrullination Activates ERK1/2 and Promotes IGF2BP1-Mediated SOX2 mRNA Stability in Endometrial Cancer

Teng Xue, Xiaoqiu Liu, Mei Zhang, Qiukai E, Shuting Liu, Maosheng Zou, Ying Li, Zhinan Ma, Yun Han, Paul Thompson, and Xuesen Zhang[*]

SUPPLEMENTAL INFORMATION

PADI2-Catalyzed MEK1 Citrullination Activates ERK1/2 and Promotes IGF2BP1-Mediated SOX2 mRNA Stability in Endometrial Cancer

Teng Xue, Xiaoqiu Liu, Mei Zhang, Qiukai E, Shuting Liu, Maosheng Zou, Ying Li, Zhinan Ma, Yun Han, Paul Thompson, Xuesen Zhang*

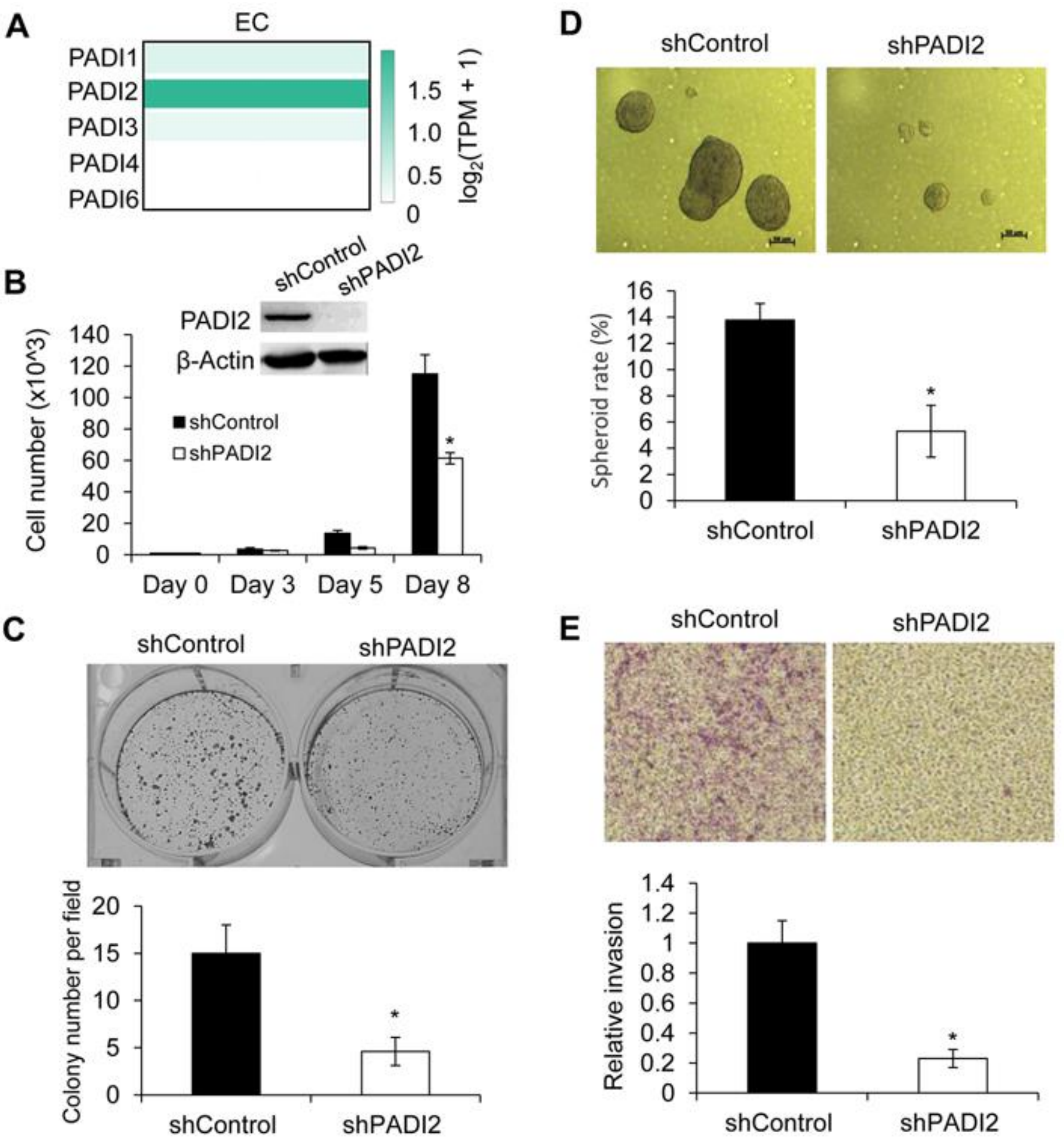

Figure S1. Related to Figure 1.

(A) The mRNA expression of 5 PADIs family members in endometrial cancer tissues in GEPIA, a web server for cancer and normal gene expression profiling and interactive analyses (http://gepia.cancer-pku.cn).

(B) PADI2 knockdown or shRNA control ECC-1 cells were cultured in regular medium, at indicated times, and cell numbers were counted under light microscope. The inserted showing the PADI2 knockdown by western blot.

(C) Representative images of crystal violet staining of cells (top) and quantification (bottom) of colony formation upon PADI2 stable knockdown in ECC-1 cells.

(D) Representative images of ECC-1 derived spheroids (diameter greater than 100 μm)

cultured at 10% FBS in concave ultra-low attachment plates (top) and quantification (bottom) upon PADI2 depletion.

(E) Representative images (top) and quantification (bottom) for transwell assay upon PADI2 depletion.

Results are presented as mean ± SEM, n = 3 (D-I). *$p < 0.05$

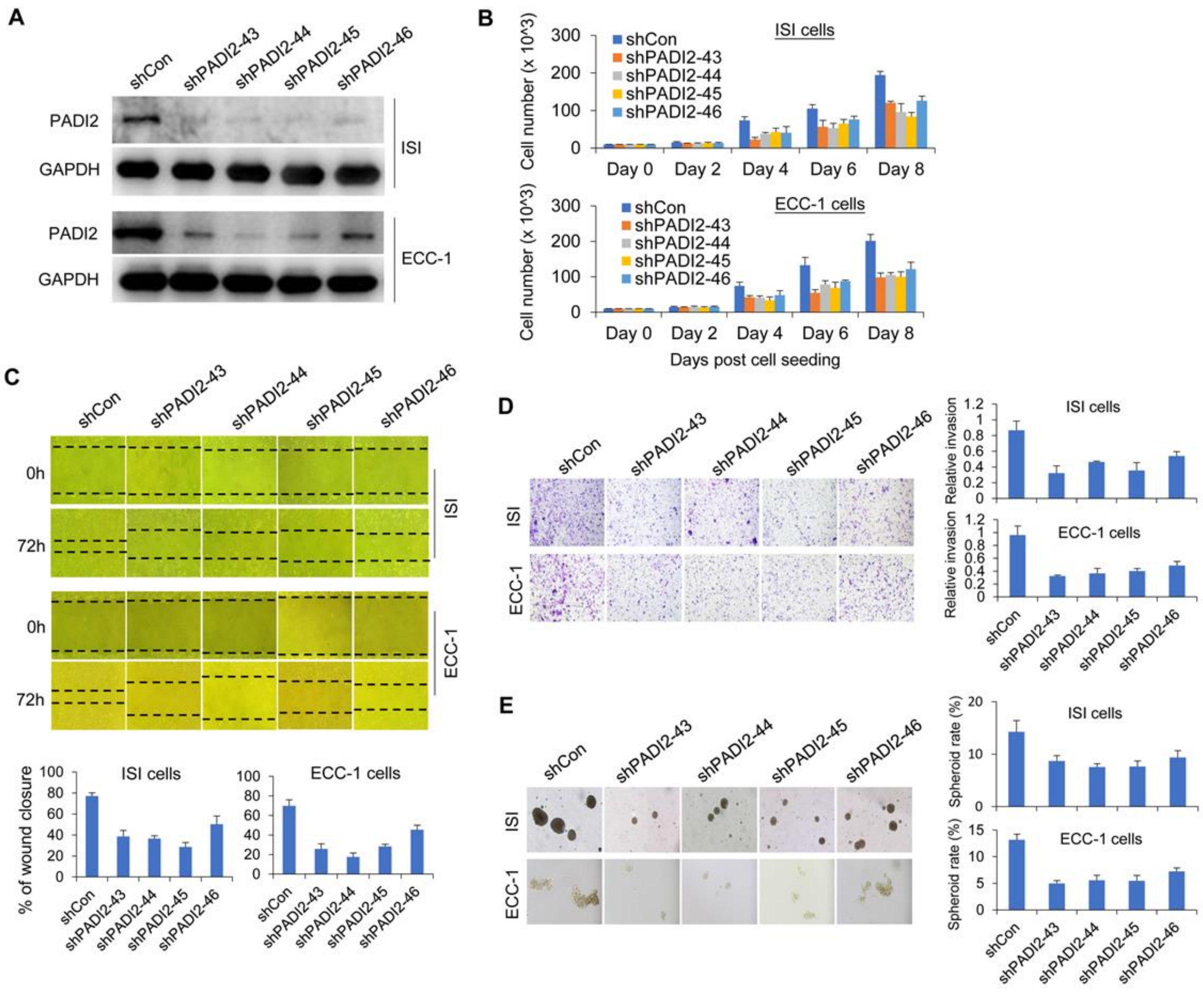

Figure S2. Related to Figures 1, 2.

(A) Western blot validation of PADI2 knockdown with 4 independent hairpins in both ISI cells and ECC-1 cells. GAPDH served as loading control.

(B) PADI2 knockdown with 4 independent hairpins or shRNA control in both ISI cells and ECC-1 cells were cultured in regular medium, at indicated times, cell numbers were counted under light microscope.

(C) Representative images at indicated hours after scratching (top) and quantification (bottom) for wound healing assay in PADI2 KD (with 4 independent hairpins) ISI or ECC-1 cells.

(D and E) Representative images (left) and quantification (right) for transwell assay (D) and spheroid formation assay (E) in PADI2 KD (with 4 independent hairpins) ISI or ECC-1 cells.

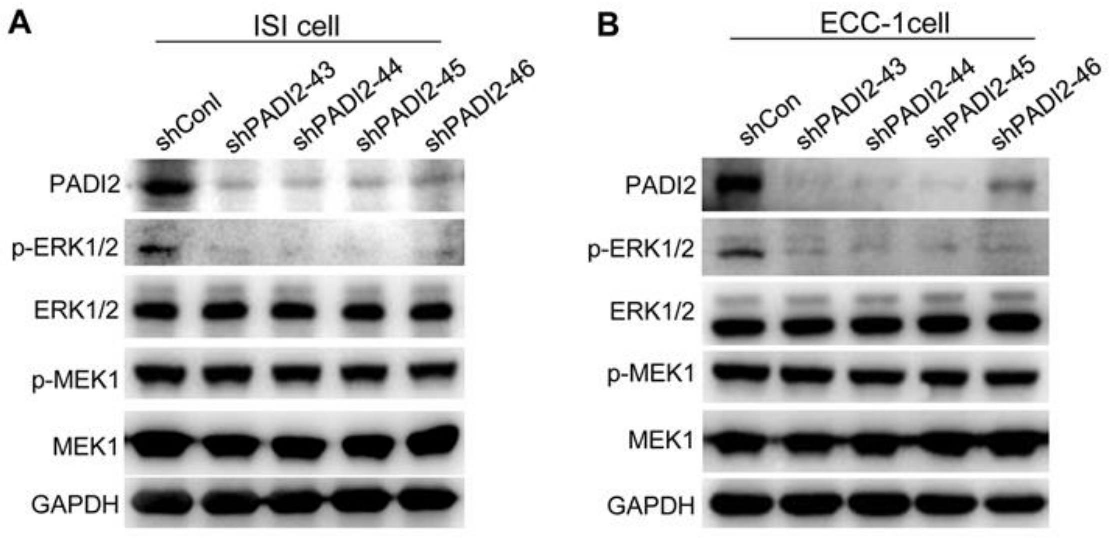

Figure S3. Related to Figure 3.

A and B: Western blot analysis of PADI2, p-ERK1/2, ERK1/2, p-MEK1, and MEK1 upon PADI2-depletion with 4 independent hairpins in ISI (A) or ECC-1 (B) cells. GAPDH used as loading control.

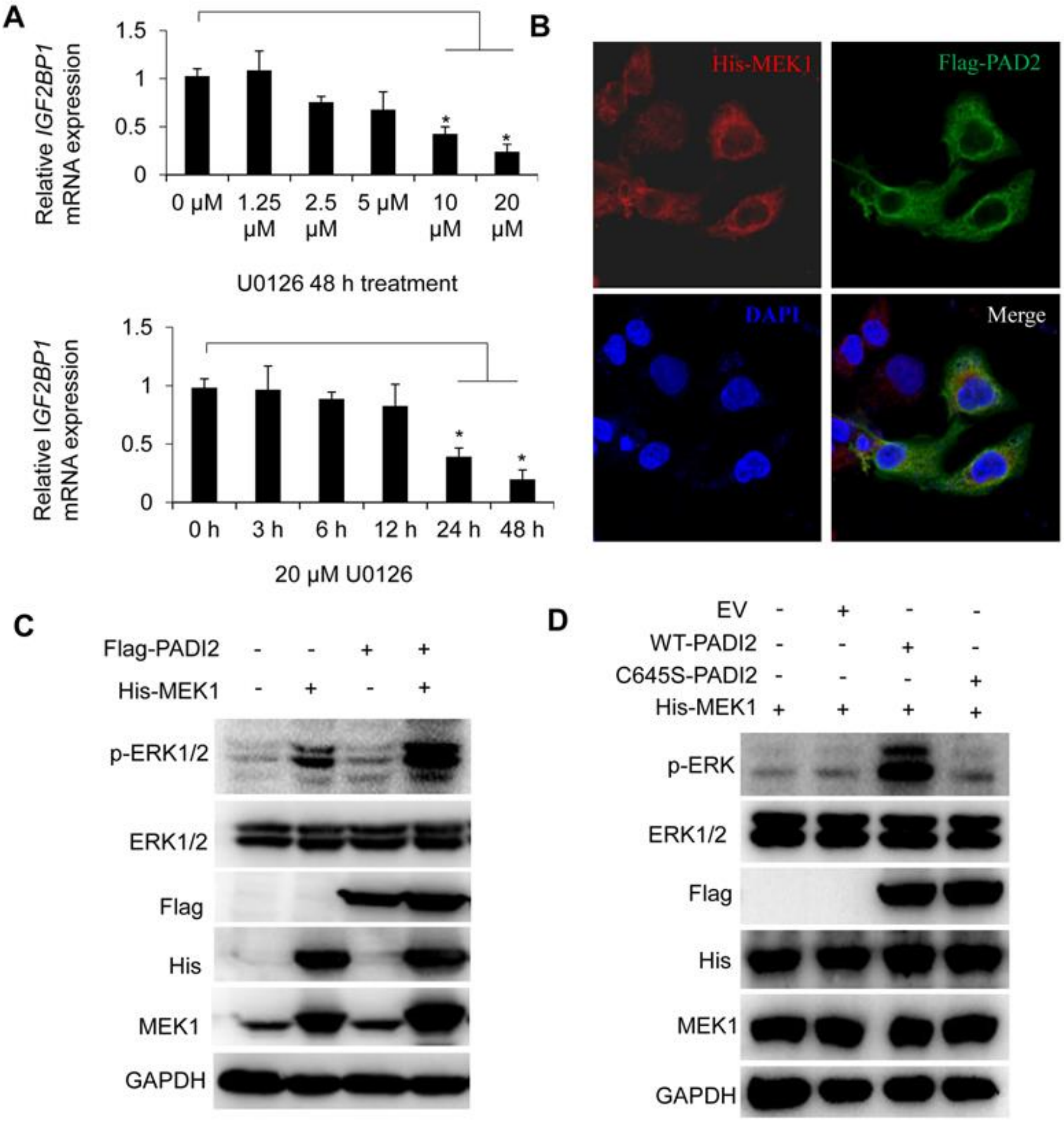

Figure S4. Related to Figure 3.

(A) The mRNA expression of *IGF2BP1* in ISI cells treated with U0126 at indicated dose for 48 hr (top), or 20 μM U0126 at indicated time (bottom).

(B) Representative immunofluorescence images of co-localization of His-tagged MEK1 (green) and Flag-PAID2 (red) in ISI cells. DAPI staining nucli.

(C and D) Western blot analysis of p-ERK1/2, ERK1/2, Flag-PADI2, His-MEK1 and GAPDH in ISI cells overexpressing Flag-tagged PADI2 and His-MEK1 (C), and in ISI cells overexpressing Flag-PADI2 (WT or Mut) together with His-MEK1 (D).

Results are presented as mean ± SEM, n = 3. $^{*}p < 0.05$

A

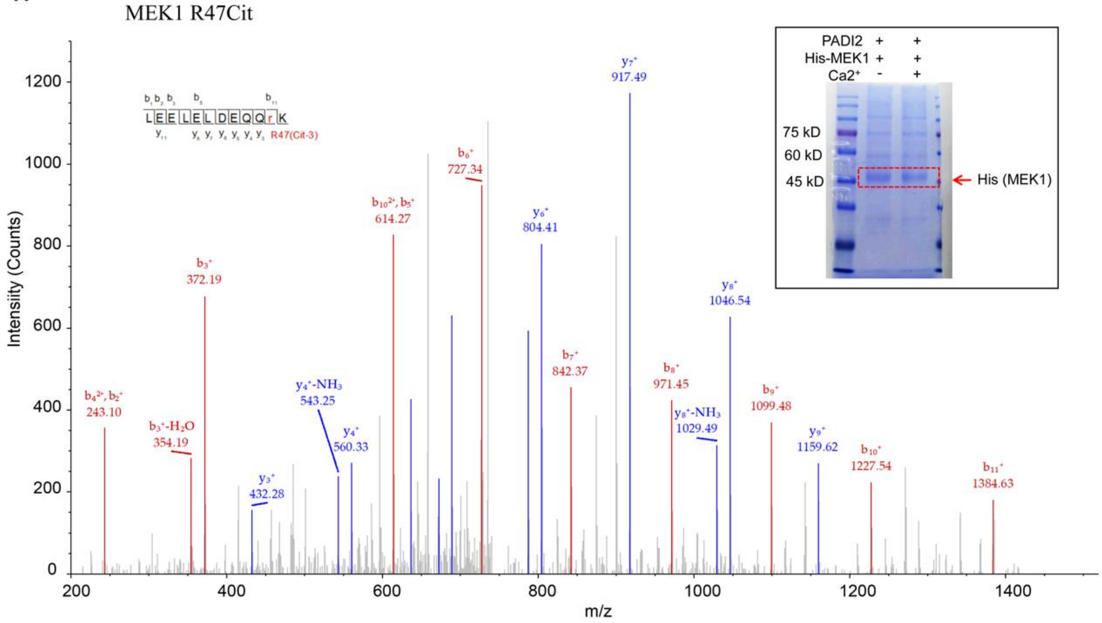
MEK1 R47Cit
LEELELDEQQrK
R47(Cit-3)
PADI2 + +
His-MEK1 + +
Ca2+ - +
75 kD
60 kD
45 kD
His (MEK1)
Intensity (Counts)
m/z
y7+ 917.49
b6+ 727.34
b10 2+, b5+ 614.27
y6+ 804.41
b3+ 372.19
y8+ 1046.54
b7+ 842.37
b8+ 971.45
y4+-NH3 543.25
b4 2+, b2+ 243.10
b3+-H2O 354.19
y4+ 560.33
y8+-NH3 1029.49
b9+ 1099.48
y9+ 1159.62
b10+ 1227.54
y3+ 432.28
b11+ 1384.63

B

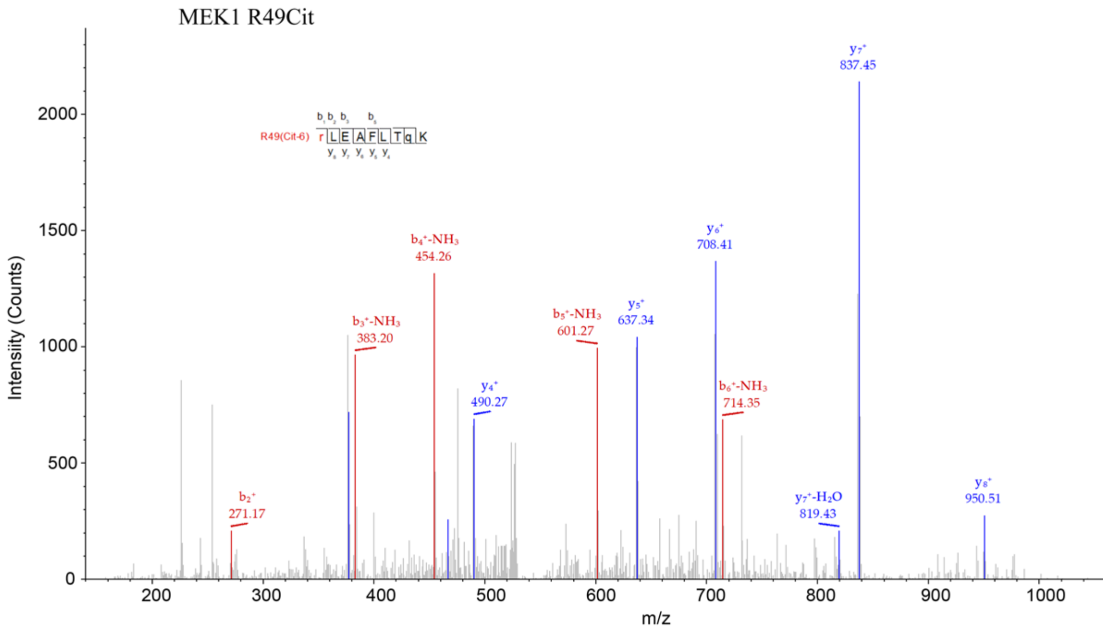
MEK1 R49Cit
R49(Cit-6) rLEAFLTqK
Intensity (Counts)
m/z
y7+ 837.45
y6+ 708.41
b4+-NH3 454.26
b3+-NH3 383.20
b5+-NH3 601.27
y5+ 637.34
y4+ 490.27
b6+-NH3 714.35
b2+ 271.17
y7+-H2O 819.43
y8+ 950.51

C

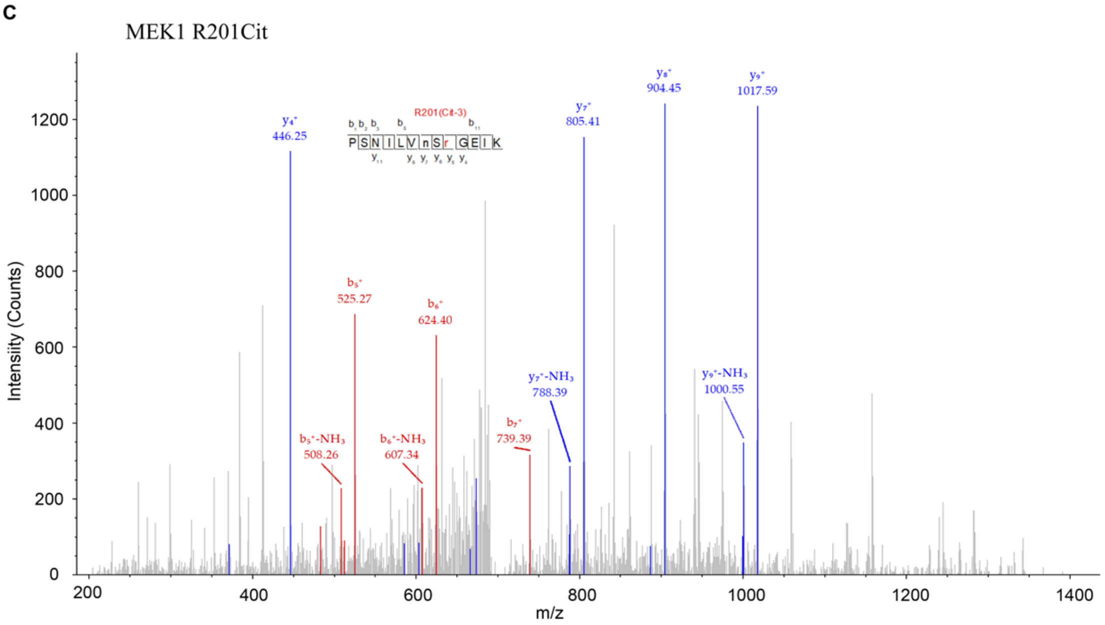

D

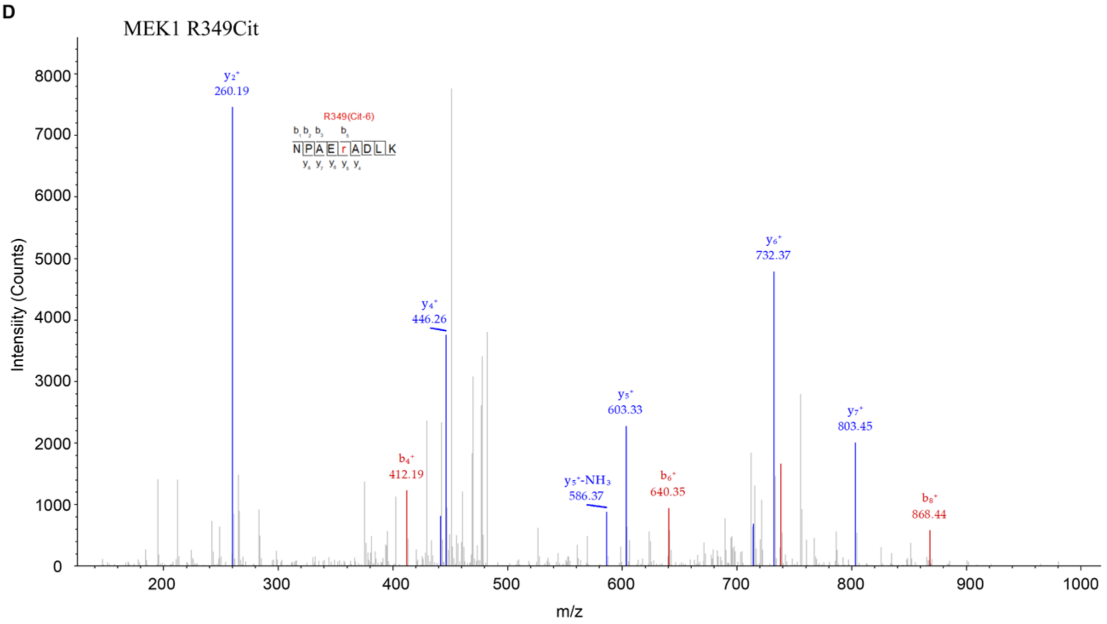

Figure S5. Related to Figure 4.

(A-D) MS/MS analysis identifies citrullination of MEK1 at R47 (A), R49 (B), R201 (C), and R349 (D). Insert in (A): Coomassie blue staining on SDS-PAGE of the His-tagged MEK1 treated with PADI2 in the presence or absence of Ca^{2+}. Red box denoting MEK1 protein with the molecular weight at 45 kDa.

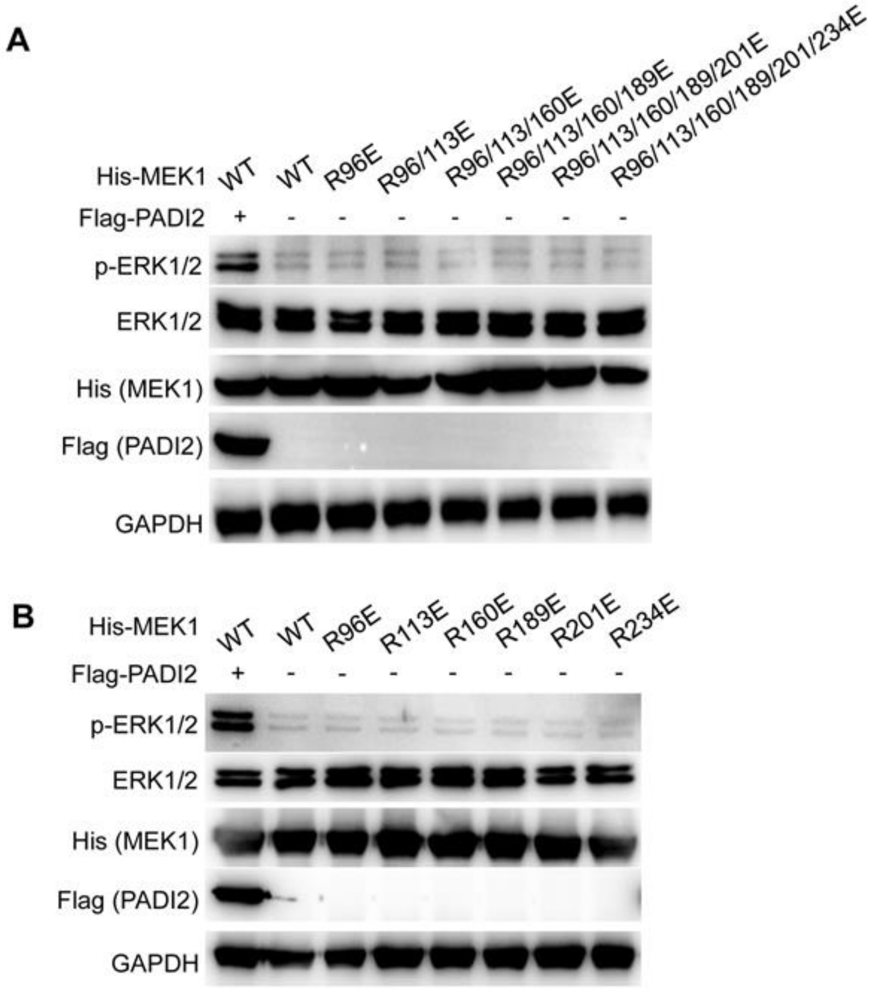

Figure S6**. Related to Figure 4.**

(A and B) Western blot analysis of p-ERK1/2 in ISI cells overexpressing a series of R/E mutants within MEK1 (A), or individual R/E mutant (B), in the absence of Flag-PADI2. EV: empty vector control.

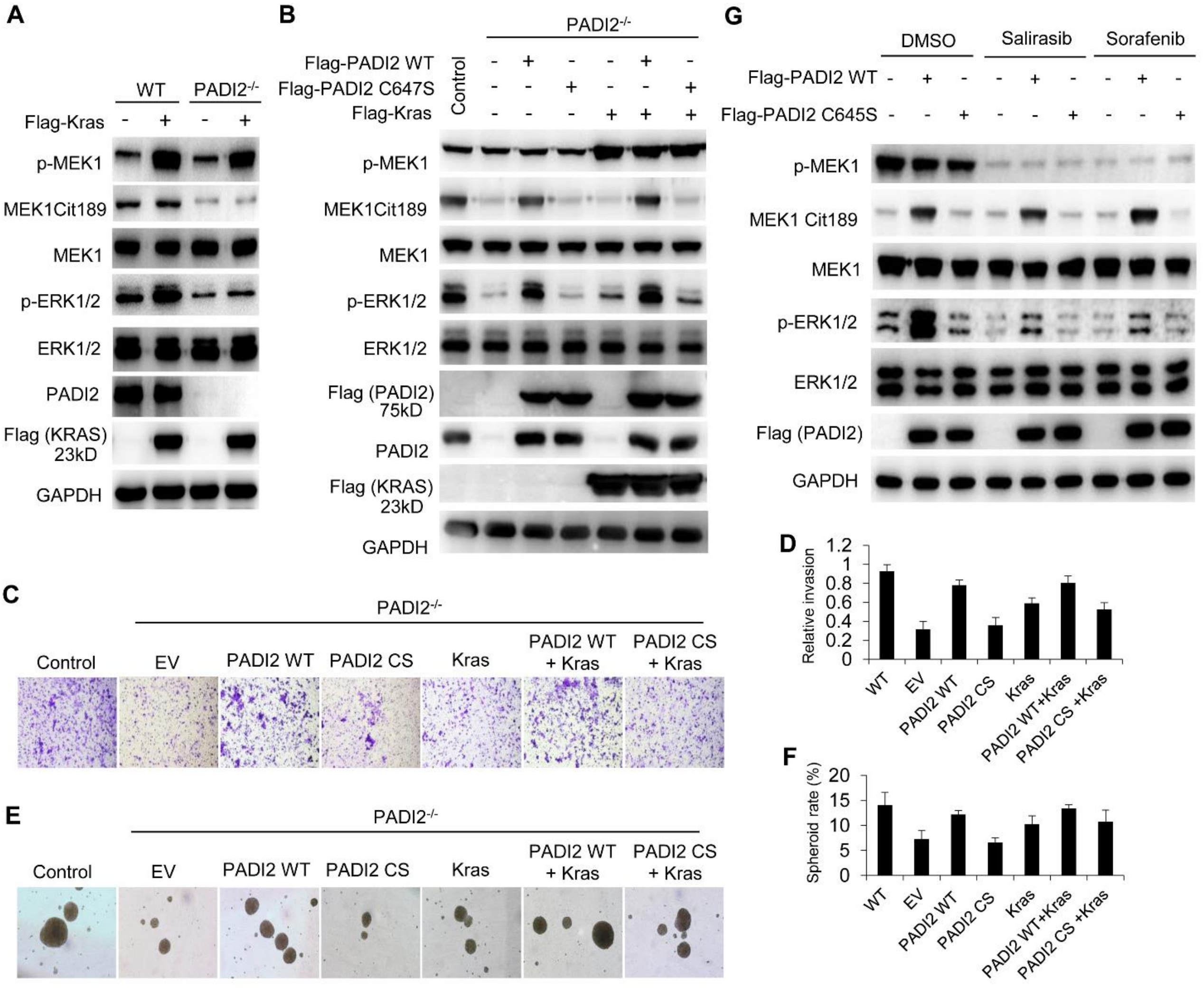

Figure S7. Related to Figure 4.

(A) Western blot analysis of p-MEK1, p-ERK1/2, and MEK1 Cit189 upon Flag-Kras overexpression in PADI2 WT or PDAI2 knockout ISI cells. GAPDH used as loading control.

(B) Western blot analysis of p-MEK1, p-ERK1/2 activation, and MEK1 Cit189 in PADI2 knockout ISI cells overexpressed with PADI2 (WT or C647S) and Flag-Kras. GAPDH used as loading control.

(C and D) Representative images (C) and quantification (D) for transwell assay in PADI2 knockout ISI cells overexpressed with PADI2 (WT or C647S) and Flag-Kras.

(E and F) Representative images (E) and quantification (F) for spheroid formation assay in PADI2 knockout ISI cells overexpressed with PADI2 (WT or C647S) and Flag-Kras.

(G) Western blot analysis of p-MEK1, p-ERK1/2 activation, and MEK1 Cit189 in HEK293 cells overexpressed with PADI2 (WT or C647S) upon Salirasib or Sorafenib treatment, compared to DMSO treatment. GAPDH used as loading control.

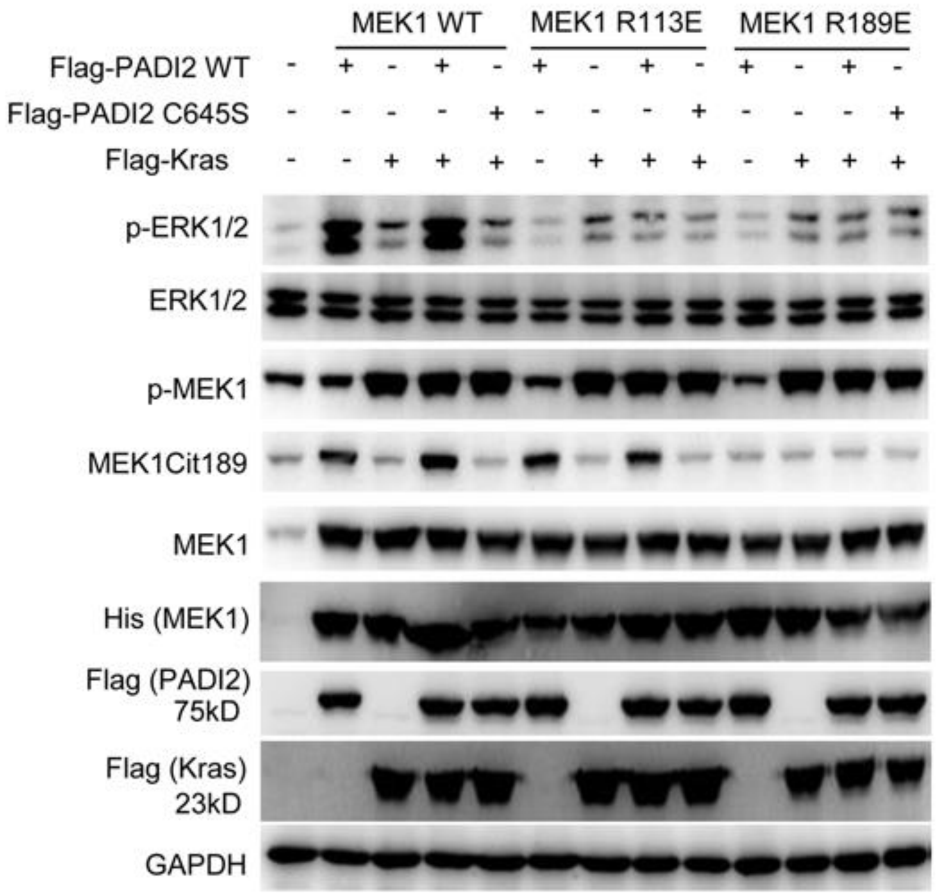

Figure S8. Related to Figure 4.

Western blot analysis of p-MEK1, p-ERK1/2 activation, and MEK1 Cit189 in HEK293 cells overexpressed with PADI2 (WT or C647S) and MEK1 WT, R113/189E mutants, in the presence of Flag-Kras. GAPDH used as loading control.

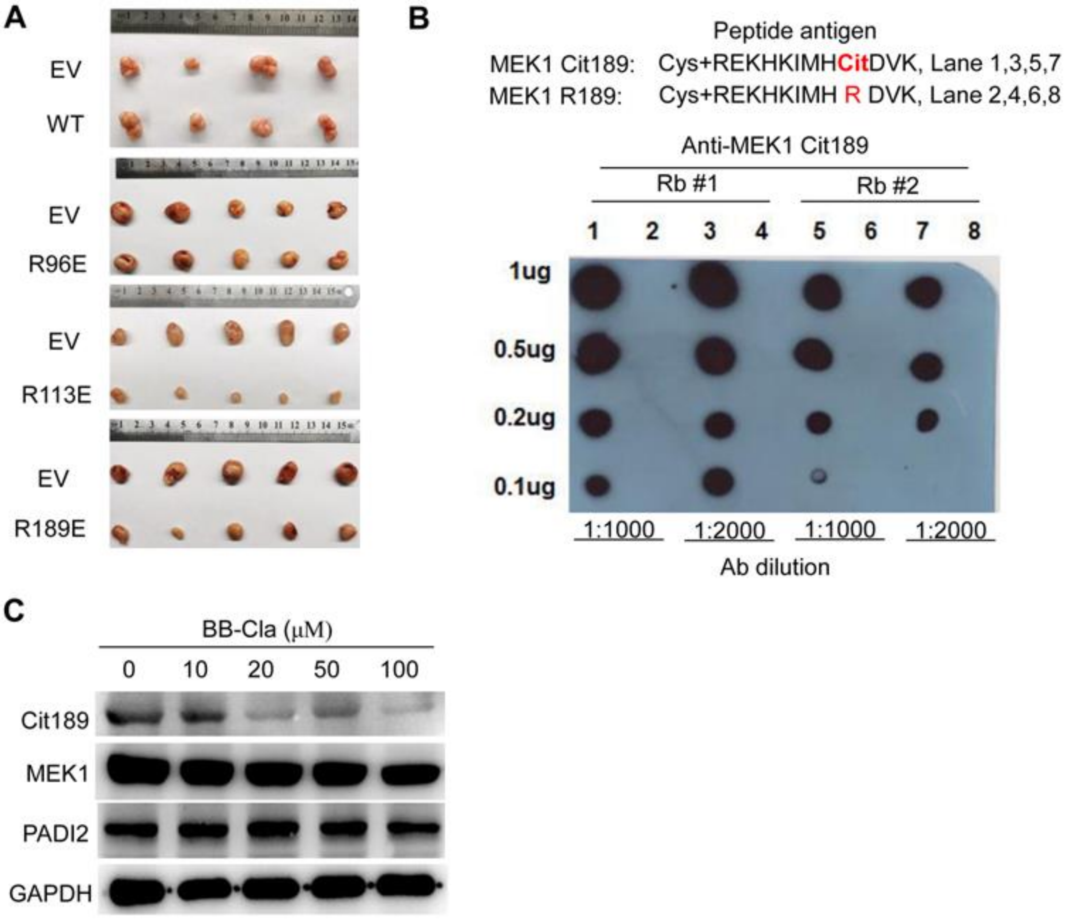

Figure S9. Related to Figure 5.

(A) Dissected tumors collected from nude mice injected with ISI cells stably overexpressing EV control on the left flank and MEK1 WT or R/E mutanton the right flank at the experimental end point.

(B) Dot blot analysis of anti-MEK1 Cit189 in detection of synthetic peptide MEK1 Cit189 and R189. Rb#1,2 representing the antibodies from the two rabbits immunized with citrullinated MEK1 peptide.

(C) Western blot analysis of Cit189, MEK1, PADI2 in ISI cells treated with BB-Cla. GAPDH served as loading control.

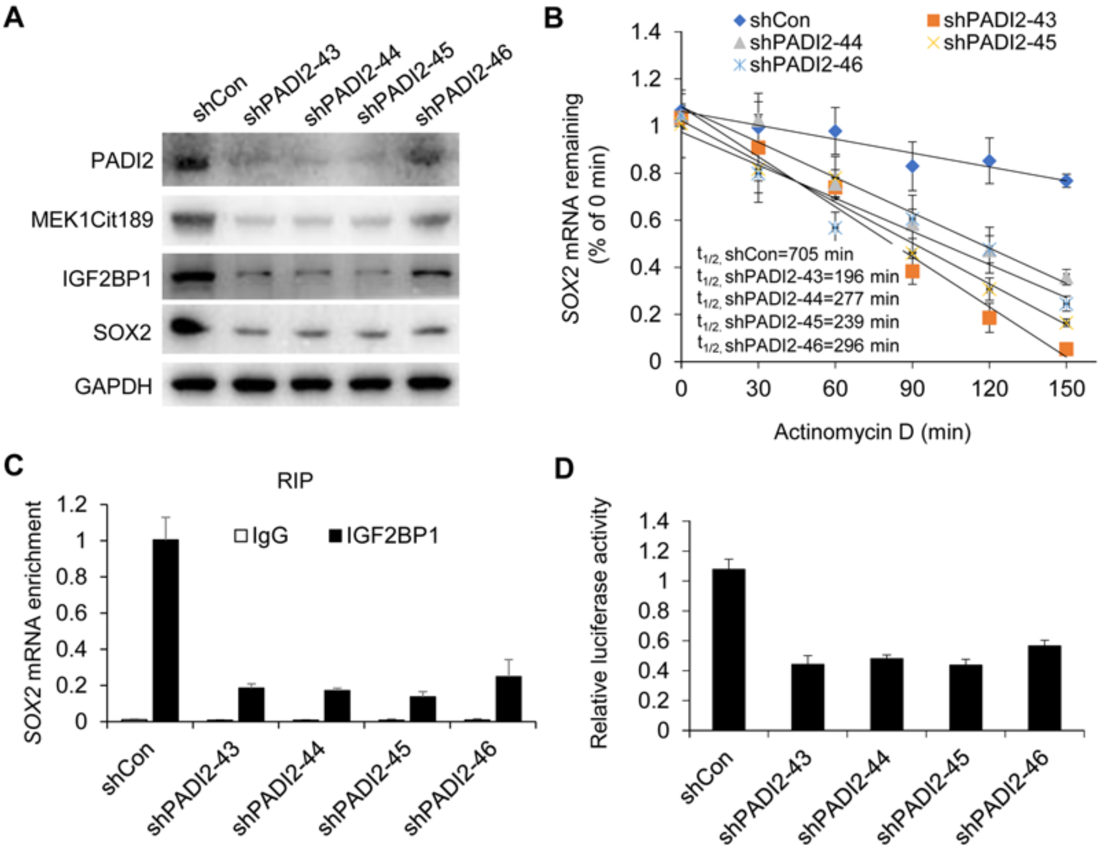

Figure S10. Related to figures 6 and 7.

(A) Western blot analysis of PADI2, MEK1 Cit189, IGF2BP1 and SOX2 upon PADI2-depletion with 4 independent hairpins in ISI cells. GAPDH used as loading control.

(B) The decay of the *SOX2* mRNA was monitored by qRT-qPCR in PADI2 knockdown with 4 independent hairpins and control ISI cells treated with actinomycin D for the indicated time. The reduction of the *SOX2* mRNA half-life by PADI2 knockdown is indicated in the graph.

(C) RIP-qPCR analysis of the enrichment of *SOX2* in PADI2 knockdown with 4 independent hairpins and control ISI cells. Differential enrichment was normalized to that of shControl and plotted as ratio. IgG used as negative control.

(D) Luciferase reporter analysis of *SOX2*-3'UTR-driven firefly luciferase reporter in PADI2-depleted with 4 independent hairpins and shRNA control ISI cells.

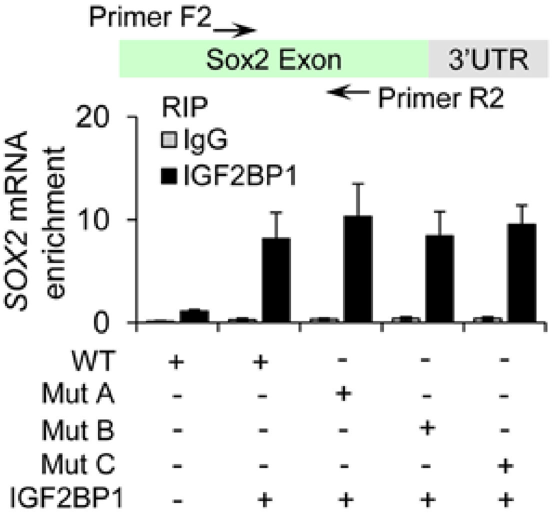

Figure S11. Related to Figure 7.

Top: Schematic representation of *SOX2* exon and respective primer pairs F2/R2 which amplifies endogenous *SOX2* segment. Bottom: RIP-qPCR analysis of the enrichment of endogenous *SOX2* RNA in ISI cells co-transfected with individual mutants shown in Figure 7E and IGF2BP1. Results are presented as mean ± SEM, n = 3. *p < 0.05.

Supplementary Table 1. The shRNA **and CRISPR sgRNA sequences**

**Gene**	**Primer**	**Sequence (5′-3′)**
ShPADI2		GTGTGCTGCATGAAGGATAAT
shPADI2-43		GCACCTTCATCGACGACATTT
shPADI2-44		CGGATACGAGATAGTTCTGTA
shPADI2-45		GTCAACTACTATGACGAGGAA
shPADI2-46		CACCTTCAAGTGGTTGCACAT
shIGF2BP1		ACGCTTAGAGATTGAACATTC
shRNA control		CAACAAGATGAAGAGCACCAA
sgPADI2-f	Forward	ACCGGGAACACGTGTGGGTGGAGG
	Reverse	AAACCCTCCACCCACACGTGTTCC
sgPADI2-r	Forward	ACCGCAGCCGGGGCCCAAACCTTC
	Reverse	AAACGAAGGTTTGGGCCCCGGCTG
SgIGF2BP1-f	Forward	ACCGGAGCGTGACCCCCGCGGACT
	Reverse	AAACAGTCCGCGGGGGTCACGTC
SgIGF2BP1-r	Forward	ACCGCCATGAACAAGCTTTACATC
	Reverse	AAACGATGTAAAGCTTGTTCATGG

Supplementary Table 2. Primers for RT-qPCR

**Gene**	**Primer**	**Sequence (5′-3′)**
PADI2	Forward	TCTCAGGCCTGGTCTCCAT
	Reverse	AAGATGGGAGTCAGGGGAAT
IGF2BP1	Forward	CAAAGGAGCCGGAAAATTCAAAT
	Reverse	CGTCTCACTCTCGGTGTTCA
TMTC1	Forward	CTGTCTTCAAGAATCGTGGACTT

	Reverse	CCGCCTCAGTATGAATAGGATGT
SPOCK1	Forward	TGCTGCACTTTACAACCACTG
	Reverse	ATCGTCTCGAAAGCGGTTCC
PKDCC	Forward	GAAGCGGAACCTCTATAATGCC
	Reverse	GGATACACTGGTACTCGGTACT
NANOG	Forward	TTTGTGGGCCTGAAGAAAACT
	Reverse	AGGGCTGTCCTGAATAAGCAG
OCT4	Forward	GCTCGAGAAGGATGTGGTCC
	Reverse	CGTTGTGCATAGTCGCTGCT
CD133	Forward	ACGCACAGGGAATGGATTGT
	Reverse	GGTTTGCACGATGCCACTTT
SOX2(F1/R1)	Forward	CAAGAAGGGCGGAAAGATCG
	Reverse	TACCGGGTTTTCTCCATGCT
SOX2(F2/R2)	Forward	AACCAGCGCATGGACAGTTA
	Reverse	CGAGCTGGTCATGGAGTTGT

Supplementary Table 3. Oligonucleotides for generating point mutation

**Gene**	**Primer**	**Sequence (5′-3′)**
R96E MEK1	Forward	GCCTGGTTATGGCTGAAAAGCTGATCCAC
	Reverse	GTGGATCAGCTTTTCAGCCATAACCAGGC
R113E MEK1	Forward	ACCAGATCATCGAGGAGCTGCAGGT
	Reverse	ACCTGCAGCTCCTCGATGATCTGGT
R160E MEK1	Forward	GAAGAAAGCTGGAGAAATTCCTGAGCAAAT
	Reverse	ATTTGCTCAGGAATTTCTCCAGCTTTCTTC

R189E MEK1	Forward	ACAAGATTATGCACGAAGATGTCAAGCCAT
	Reverse	ATGGCTTGACATCTTCGTGCATAATCTTGT
R201E MEK1	Forward	CATTCTAGTGAACTCAGAAGGGGAGATCAAACT
	Reverse	AGTTTGATCTCCCCTTCTGAGTTCACTAGAATG
R234E MEK1	Forward	CATGTCGCCTGAGGAACTCCAGGGGACTC
	Reverse	GAGTCCCCTGGAGTTCCTCAGGCGACATG
R113A MEK1	Forward	ACCAGATCATCGCGGAGCTGCAGGT
	Reverse	ACCTGCAGCTCCGCGATGATCTGGT
R113K MEK1	Forward	ACCAGATCATCAAGGAGCTGCAGGT
	Reverse	ACCTGCAGCTCCTTGATGATCTGGT
R189A MEK1	Forward	ACAAGATTATGCACGCAGATGTCAAGCCAT
	Reverse	ATGGCTTGACATCTGCGTGCATAATCTTGT
R189K MEK1	Forward	ACAAGATTATGCACAAAGATGTCAAGCCAT
	Reverse	ATGGCTTGACATCTTTGTGCATAATCTTGT
R201A MEK1	Forward	CATTCTAGTGAACTCAGCTGGGGAGATCAAACT
	Reverse	AGTTTGATCTCCCCAGCTGAGTTCACTAGAATG
R201K MEK1	Forward	CATTCTAGTGAACTCAAAAGGGGAGA

		TCAAACT
	Reverse	AGTTTGATCTCCCCTTTTGAGTTCACTAGAATG
R234A MEK1	Forward	CATGTCGCCTGAGGCACTCCAGGGGACTC
	Reverse	GAGTCCCCTGGAGTGCCTCAGGCGACATG
R234K MEK1	Forward	CATGTCGCCTGAGAAACTCCAGGGGACTC
	Reverse	GAGTCCCCTGGAGTTTCTCAGGCGACATG
Mut A SOX2 3'UTR	Forward	ACCGGGCCGGGCAGCGAACTGGAGGGGGGAGAAATTTTCAAA
	Reverse	CAGTTCGCTGCCCGGCCCGGTACCACCGGTGAATTCTCTTGA
Mut B SOX2 3'UTR	Forward	ATGAGAGAGATCCTGGGCTTCTTTTTGG
	Reverse	CCAAAAAGAAGCCCAGGATCTCTCTCAT
Mut C SOX2 3'UTR	Forward	CTTTTTGGGGGGCTATTTTTGTACAGA
	Reverse	TCTGTACAAAAATAGCCCCCCAAAAAG

Supplementary Table 4. Antibodies

Antibodies	Source	Identifier
Rabbit anti-PADI2	Proteintech	Cat# 12110-1-AP
Rabbit anti-p-p44/42 MAPK(T202/Y204)	Cell Signaling Technology	Cat# 4370

Rabbit anti-p44/42 MAPK(ERK1/2)	Cell Signaling Technology	Cat# 4695
Rabbit anti-p-MEK1/2 (S217/221)	Cell Signaling Technology	Cat# 9154
Rabbit anti-MEK1	Cell Signaling Technology	Cat# 8727
Rabbit anti-p-AKT (S473)	Cell Signaling Technology	Cat# 4060
Rabbit anti-AKT	Cell Signaling Technology	Cat# 9272
Rabbit anti-p-p38 MAPK (T180/Y182)	Cell Signaling Technology	Cat# 4511
Rabbit anti-P38 MAPK	Cell Signaling Technology	Cat# 8690
Rabbit anti-p-RPS6 (S235/236)	Cell Signaling Technology	Cat# 2211
Rabbit anti-RPS6	Cell Signaling Technology	Cat# 2217
Rabbit anti-IGF2BP1	Abcam	Cat# ab82968
Rabbit anti-SOX2	Bioworld Technology	Cat# BS6161
Mouse anti-His	Bioworld Technology	Cat# AP0032M
Rabbit anti-His	Cell Signaling Technology	Cat# 12698
Mouse anti-GAPDH	Bioworld Technology	Cat# AP0063
Mouse anti-β-Actin	Abcam	Cat# ab6276
Mouse anti-Flag	Sigma	Cat# F1804
Mouse anti-PCNA	Abcam	Cat# ab29
Rabbit anti-MEK1 Cit189	AtaGenix Biotechnology	Not commercially available

Goat anti-rabbit IgG, HRP-linked	Cell Signaling Technology	Cat# 7074
Horse anti-mouse IgG, HRP-linked	Cell Signaling Technology	Cat# 7076
Goat anti-Mouse IgG, Alexa Fluor 488 linked	Invitrogen	Cat# A-11001
Goat anti-Rabbit IgG, Alexa Fluor 546 linked	Invitrogen	Cat# A-11035
Normal Mouse IgG	Santa Cruz Biotechnology	Cat# sc-2025
Normal Rabbit IgG	Millipore	Cat# 12-370
